# Overcoming platinum resistance by the disulfiram metabolite CuET in novel preclinical models of human carcinomas

**DOI:** 10.1002/1878-0261.70356

**Published:** 2026-11-04

**Authors:** Stefanos Arampatzis, Tereza Buchtová, Jirina Bartkova, Marie Lund Bay, Christoffer Lynge Halvorsen, Zuzana Machacova, Zdenek Skrott, Martin Mistrik, Ekaterina Dulina, Miguel Andujar‐Sanchez, Estrid Høgdall, Tine Henrichsen Schnack, Claus Høgdall, Berit Jul Mosgaard, Marie Boennelycke, Jacob Brink Laursen, Allan Jensen, Susanne K. Kjær, Tuula Kallunki, Robert Strauss, Jiri Bartek

**Affiliations:** ^1^ Genome Integrity Group Danish Cancer Institute, Danish Cancer Society Copenhagen Denmark; ^2^ Division of Genome Biology, Department of Medical Biochemistry and Biophysics, Science for Life Laboratory Karolinska Institute Stockholm Sweden; ^3^ Cancer Invasion and Resistance Group Danish Cancer Institute, Danish Cancer Society Copenhagen Denmark; ^4^ Faculty of Medicine and Dentistry Institute of Molecular and Translational Medicine, Palacky University Olomouc Czech Republic; ^5^ Department of Pathology Complejo Hospitalario Universitario Insular Las Palmas Gran Canaria Spain; ^6^ Molecular Unit, Department of Pathology Copenhagen University Hospital, Herlev and Gentofte Hospital Denmark; ^7^ Department of Gynaecology Copenhagen University Hospital Rigshospitalet Denmark; ^8^ Department of Clinical Medicine University of Copenhagen Denmark; ^9^ Department of Pathology Copenhagen University Hospital Rigshospitalet Denmark; ^10^ Virus, Lifestyle and Genes Group Danish Cancer Institute, Danish Cancer Society Copenhagen Denmark; ^11^ Translational and Clinical Pharmacology, Department of Drug Design and Pharmacology University of Copenhagen Denmark; ^12^ Division of Cancer Research, Ninewells Hospital and Medical School University of Dundee UK

**Keywords:** biomarkers, breast and ovarian cancer, disulfiram metabolite, lung, metallothionein, platinum resistance

## Abstract

Overcoming resistance to standard‐of‐care chemotherapy represents an unmet need in oncology. To help address this challenge, here we established and characterized a panel of human cisplatin‐resistant lung and breast cancer cell lines and patient‐derived organoids from high‐grade serous ovarian carcinomas (HGSOC), and show that platinum resistance can be overcome by treatment with diethyldithiocarbamate‐copper (CuET), a metabolite of the alcohol‐aversion drug disulfiram. All platinum‐resistant models remained sensitive to CuET, consistent with distinct cytotoxicity mechanisms: proteotoxic stress for CuET and DNA damage for cisplatin, respectively. Platinum adduct and DNA damage response analyses suggested diverse mechanisms of cisplatin resistance among our models. Considering potential CuET‐based therapies, our gene knock‐down and functional assays in preclinical models, complemented with immunohistochemical analysis of tissue samples from cohorts of triple‐negative breast cancer and HGSOC patients indicate that levels of metallothioneins, rather than NPL4 (the molecular target of CuET) warrant further evaluation as candidate biomarkers of CuET sensitivity. This dataset provides a resource of platinum‐resistant models of common carcinomas, identifies CuET as a compound overcoming such resistance and provides leads for predictive biomarker discovery, with potential clinical implications in oncology.

## Graphical abstract

Cisplatin‐resistant cancer cells survive DNA‐damaging platinum‐based treatment yet remain sensitive to CuET‐induced proteotoxic stress. The distinct mechanism of action allows CuET to overcome and eliminate cisplatin‐resistant tumour cells, unless highly expressed metallothioneins (MTs) interfere with CuET, thereby highlighting MT levels as candidate predictive biomarkers of CuET sensitivity. Created in BioRender. Buchtová, T. (2026) https://BioRender.com/wugac2r.

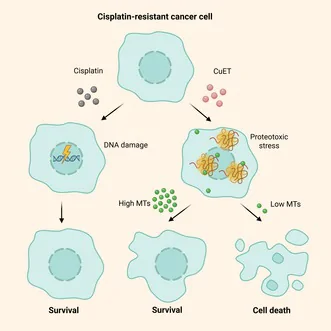

**Abbreviations**
ATCC: American Tissue Culture Collection
ATR: Ataxia Telangiectasia and Rad3‐related
cisR: cisplatin‐resistant
CuET: bis‐(diethyldithiocarbamate)‐copper
DSF: disulfiram
ER: oestrogen receptor
FDA: Food and Drug Administration
HER2: human epidermal growth factor receptor 2
LUAD: lung adenocarcinoma
NPL4: nuclear protein localization protein 4
NSCLC: non‐small cell lung cancer
NT: nontreated
PR: progesterone receptor
RI: resistance index
ROS: reactive oxygen species
TNBC: triple‐negative breast cancer
VCP: valosin‐containing protein
WT: wild‐type

## 1 Introduction

Platinum‐based drugs, particularly cisplatin and later carboplatin, have long been used as part of standard‐of‐care chemotherapy for diverse types of human malignancies, including such common tumour types as carcinomas of the lung, breast, ovary or urinary bladder [1, 2]. Upon entering the cell, cisplatin undergoes activation in the cytoplasm, turning into an electrophilic molecule that mainly exerts its anticancer potential through platinum adducts inducing cytotoxic inter‐ and intra‐strand DNA crosslinks. On the contrary, the main mechanism through which carboplatin induces cancer cell toxicity is interference with ribosome biogenesis [3], a process that is commonly aberrantly enhanced in tumour cells, thereby providing a targetable vulnerability [4].

Despite the recent advances in cancer research and continuous development and improvements of cancer treatment modalities and biological treatments, including surgery, radiotherapy, and immunotherapy, chemotherapy remains a cornerstone of therapy even in contemporary oncology. Chemotherapy in general, and cisplatin in particular, is also used as an adjuvant or neoadjuvant treatment, and even in palliative care [5]. However, chemotherapy efficacy is limited by the development of tumour cell chemoresistance, a major challenge which eventually occurs in the majority of patients with advanced cancer, and to which up to 80–90% of cancer‐related deaths appear to be attributable [6].

Platinum resistance can be acquired via different molecular mechanisms, the most common of which reflect selection of cancer cells with adaptive pro‐survival changes in DNA damage and other stress‐response pathways, such as the nucleotide excision repair and the mismatch repair which are predominantly responsible for repairing the platinum‐induced DNA lesions. Other factors contributing to platinum resistance include reduced uptake or enhanced efflux of the drugs by the cells [7, 8], increased metabolic detoxification, aberrations in the signalling and/or effector pathways responsible for tumour cell death, as well as epigenetic deregulation of relevant genes via DNA methylation and miRNAs [8].

Overcoming chemotherapy resistance is therefore critical for improving clinical outcomes in oncology, prevention of tumour recurrence and reduction of the harmful side effects caused by prolonged exposure of patients' normal tissues to cytotoxic drugs. Different approaches may help address this challenge, such as combinatorial therapies (allowing lower doses of a given chemotherapeutic agent), development of new targeted and biological therapy strategies, enhanced drug delivery systems, inhibition of drug efflux and modification of the tumour microenvironment [9]. The downside of most such approaches is that they require the development of new drug entities, a process that involves lengthy, costly, complex and uncertain preclinical development and clinical trials. An alternative strategy to overcome chemoresistance is drug repurposing of already approved drugs [10]. Following the latter line of thought, here we take advantage of our studies on disulfiram (DSF), specifically its metabolite CuET (bis‐diethyldithiocarbamate‐copper complex), which we discovered and showed to be primarily responsible for DSF's anticancer activity [11, 12]. DSF was the first approved drug for the treatment of alcohol abuse in 1951, and later also applied in treating cocaine addiction, besides displaying some antimicrobial, antiparasitic and anti‐inflammatory effects [13].

Preclinical cancer research has already shown that DSF is cytotoxic for cells of lung, breast, ovarian and prostate carcinomas as well as multiple myeloma [14, 15]. Mechanistically, we reported that CuET disables the p97/VCP (valosin‐containing protein) protein turnover network upstream of the proteasome, via aggregation and thereby functional inhibition of NPL4, a key adaptor protein of the p97 segregase complex [12]. Moreover, we reported that CuET also impairs protein translation at the ribosomes and the function of the p53 tumour suppressor [16], as well as can induce, in a context‐dependent manner, replication stress and blockade of ATR signalling [17].

Given the urgent need for better understanding and overcoming cancer chemoresistance, here we wished to establish and characterize a series of human platinum‐resistant lung and breast cancer cell lines and patient‐derived organoids from high‐grade serous ovarian carcinomas (HGSOC) and examine whether CuET could be used as an agent also against such chemoresistant models. Moreover, we wished to provide mechanistic insights into responses of these newly established models to cisplatin and CuET, and address the notion of potential sensitivity vs. resistance to CuET treatment in both preclinical experiments and by examination of candidate predictive biomarkers in clinical specimens from cohorts of HGSOC and triple‐negative breast carcinomas.

## 2 Materials and methods

### 2.1 Cell lines

All cell lines used have been mycoplasma free, all purchased from the American Tissue Culture Collection (ATCC) and authenticated by genetic fingerprinting. Human non‐small‐cell lung cancer cell lines H1792 (NCI‐H1792; RRID:CVCL_1495), HCC4006 (RRID:CVCL_1269), A549 (A‐549; RRID:CVCL_0023) and H1944 (NCI‐H1944; RRID:CVCL_1508) were cultured in RPMI Medium 1640 (61 870; Gibco, Waltham, MA, USA). Human triple‐negative breast cancer cell lines MDA‐MB‐231 (RRID:CVCL_0062) and MDA‐MB‐468 (RRID:CVCL_0419) and high‐grade serous ovarian cancer cell line OVCAR‐5 (RRID:CVCL_1628) were grown in Dulbecco's Modified Eagle Medium (31 966; Gibco, USA). Both types of media were supplemented with 10% fetal bovine serum (A5256701; Gibco, USA) and 1% penicillin/streptomycin (15 140; Gibco, USA).

### 2.2 Cytotoxicity assays

Three thousand cells were plated in a 96‐well plate. Forty‐eight hours later, cells were treated with CuET or cisplatin using the Agilent Bravo SRT. The highest final concentration used was 2500 nm for CuET and 50 μm for cisplatin, while the remaining eight concentrations were 1 : 2 serial step dilutions of the previous higher concentration. Ninety‐six hours after treatment, cells were stained with SYTOX™ Deep Red Nucleic Acid Stain (S11381; Thermo Fisher Scientific, Waltham, MA, USA) at a final concentration of 0.05 μm and Hoechst (33 342; Thermo Fisher Scientific, USA) at a final concentration of 6 μm. After incubating for 30 min at 37 °C in the dark, the plate was imaged on the scanR High‐Content Screening Station. On the scanR Analysis software, live cells were gated as Hoechst positive and SYTOX™ negative and were used for the calculation of IC_50_ values.

As a complementary approach, the cytotoxicity was evaluated using crystal violet assay (used to obtain the data shown in Fig. 7B,C), performed using a modified version of a previously described protocol [7, 18], as follows: 10000 cells per well were seeded in 96‐well plates. After 24 h, the cells were treated with the indicated concentrations of CuET and incubated for an additional 48 h. The culture medium was then removed, and the cells were fixed with ice‐cold 70% ethanol and stained with 1% crystal violet in 20% methanol for 15 min. The staining solution was removed, and the plates were thoroughly washed with water, dried, and scanned. For quantitative analysis, the cell‐bound dye was dissolved in 10% acetic acid, and the absorbance was measured at 570 nm using an InfiniteM200 PRO microplate reader (Tecan). The results are expressed relative to those of control cells, presented as pooled data from three independent biological experiments.

### 2.3 Immunofluorescence staining and quantitative microscopy

Cells were grown on coverslips or in a PhenoPlate™ 96‐well microplate (6 055 300; Revvity, Waltham, MA, USA). Forty‐eight hours after plating, cells were treated with the drugs for 4 h. Afterwards, cells were fixed with 4% formaldehyde (VWRC9713.1000, PA, USA) for 15 min, permeabilized with 0.5% Triton X‐100 (T8787; Merck, Rahway, NJ, USA) for 8 min, and blocked with DMEM supplemented with 5% fetal bovine serum for 1 h. Next, the samples were incubated with the primary antibodies overnight at 4 °C. The following day, the samples were incubated with the secondary antibodies and DAPI (62 247; Thermo Fisher Scientific, USA) for 1 h at room temperature. For mounting, Fluoromount‐G® (SouthernBiotech, Birmingham, AL, USA) was used. The cell images were then visualized with the scanR High‐Content Screening Station, and the quantitative analysis was performed with the ScanR Analysis software.

For staining with the antibody against the K48 ubiquitinated proteins, cells were pre‐extracted with 0.1% Triton X‐100 for 1 min before fixation. For the detection of the cisplatin induced CDDP‐DNA adducts, we employed the previously published methodology [7].

### 2.4 Quantitative polymerase chain reaction (qPCR)

Cells were plated in 60‐mm diameter plates, with 300 000 cells per plate. Forty‐eight hours after seeding the plates, cells were treated for 4 h with the desired drugs. Total RNA was then purified from the harvested samples using the RNeasy Mini Kit (74 104; Qiagen, Hilden, Germany) according to the manufacturer's instructions. Quantification and quality control of the isolated samples were performed using the NanoDrop™ 2000c Spectrophotometer (Thermo Scientific, USA). Two micrograms of total RNA were used to synthesize single‐stranded cDNA using the High‐Capacity cDNA Reverse Transcription Kit (Applied Biosystems, Foster City, CA, USA) following the manufacturer's instructions and using the ProFlex PCR System (Thermo Fisher Scientific, USA). Fifteen nanograms of cDNA were used to perform the qPCR, using the Fast SYBR™ Green Master Mix (4 385 612; Thermo Fisher Scientific, USA). Samples were loaded in 96‐Well Reaction Plates (4 366 932; Applied Biosystems), and the reaction was performed in the 7500 Fast Real‐Time PCR System (Applied Biosystems). Delta–delta CT analysis was performed for the calculation of gene expression. The primers used were published previously [19].

### 2.5 RNA interference

A total of 300 000 cells were plated in 6‐well plates. Twenty‐four hours later, cells were transfected with the siRNA for *MTF1* with Lipofectamine RNAiMAX (13 778 075; Invitrogen, Waltham, MA, USA) according to the manufacturer's instructions. The final concentration used for the siRNA was 10 μM/mL. Cells were reseeded 24 h later in 96‐well plates for the cytotoxicity assay and 60‐mm plates for the qPCR and the ICC assays. Each of the subsequent functional assays was performed as described in the respective subsection of the Materials and Methods section. *MTF1* siRNA were Silencer™ Select Pre‐Designed siRNA (Ambion, ID s9027, cat. no.: 4394241) and siRNA Oligo Duplex (OriGene, cat. no.: SR302991 with SR30004, Trilencer‐27 Universal Scrambled Negative Control siRNA Duplex), siLuciferase, used as a negative control, was ordered from Eurofins Genomics (5′‐CGUACGCGGAAUACUUCGA‐3′).

### 2.6 Immunoperoxidase cyto‐ and histo‐chemistry

Cells were cultured on glass coverslips in 60‐mm diameter dishes in standard media and the appropriate treatments. Coverslips were washed in phosphate‐buffered saline (PBS), fixed in 4% formaldehyde for 10 min and washed 3× in PBS. The coverslips with fixed cells or the deparaffinized tissue sections of formalin‐fixed clinical samples from the well‐established cohorts [20, 21, 22, 23, 24] of human triple‐negative breast carcinomas (*N* = 60) and HGSOC (*N* = 51) were then incubated with the appropriate primary antibodies against metallothioneins MT1 and MT2 (mouse monoclonal from Novus Biologicals, Centennial, CO, USA, no.: NBP1‐97493, diluted 1 : 500) and NPL4 (rabbit polyclonal from Atlas Antibodies, no.: HPA 023295, diluted 1 : 250), respectively, overnight in a wet chamber at 4 °C. Following several washes, the Vectastain Elite kit (Vector Laboratories, Burlingame, CA, USA) was employed as the secondary reagent to detect the bound primary antibodies. For the visualization steps, a sensitive, previously applied indirect immunoperoxidase staining method with chromogen metal enhancement and without nuclear counterstaining was used [20, 25], to allow for sensitive detection of the examined proteins. Positive (anti‐TPR rabbit antibody, Sigma‐Aldrich, no.: HPA019661, diluted 1 : 1000) and negative (nonimmune serum) controls were included in all experiments. Immunohistochemical staining was evaluated by an experienced oncopathologist. NPL4 staining intensity was scored as normal (comparable staining intensity to that seen in the normal cells present on the same tissue section) or high (aberrantly higher than that in the surrounding normal cells). For metallothionein expression patterns, we followed the reported scoring categories according to fraction of stained cancer cells [26], as follows: negative (below 1% + ve tumour cells), low (1–5% + ve tumour cells), medium (6–50% + ve tumour cells) or high (over 50% + ve tumour cells). Representative images of the obtained immunohistochemical staining patterns are shown in Fig. 8.

### 2.7 Western blotting

Protein lysates were separated by SDS/PAGE on commercial Bis‐Tris precast (4–12%, Thermo Fischer) or hand‐cast gels. Separated proteins were transferred onto nitrocellulose membrane and the studied proteins detected by the relevant antibodies, essentially as described [20, 25]. The membrane was blocked in 5% milk in PBS‐Tween‐20, incubated in primary antibodies overnight at 4 °C, and for 1 h in secondary antibodies at room temperature. Bound antibodies were detected using substrate (32132; Thermo Fischer, USA) and ChemiDoc instrument (Bio‐Rad, Hercules, CA, USA).

### 2.8 Tumour organoids

Patient‐derived tumour organoids (PDOs) were established as long‐term cultures from biopsies obtained from HGSOC tumour tissues of patients undergoing primary surgery before initiating chemotherapy. The PDOs used in this current study are part of a larger collection of a patient cohort recruited from Rigshospitalet (Copenhagen, Denmark) with the following exclusion criteria: lack of consent, lack of understanding information material, < 18 years of age, non‐HGSOC, other active cancer. Permission to establish and study the organoids from HGSOC tumours of consented patients (ethical permission no.: H‐21071553; OVACAN) was received from the ethical committee of the Capital Region, Denmark. PDO cultures were established according to published protocol [27] and used for experiments at passage > 11. For testing sensitivity to carboplatin, cisplatin and CuET, PDOs were seeded in Screenstar microwell 96‐well plates (Greiner Bio‐One) in 5.95 mg/mL basal membrane extract (BME) matrix (Cultrex PathClear Reduced Growth Factor BME, Type 2 from Bio‐Techne®). Three days before drug addition, the medium was changed to Human Plasma‐Like Medium (Gibco™). Drugs were added in twofold dilution series along with vehicle negative controls (water for cisplatin and carboplatin, DMSO for CuET) 96 h before evaluating cell death response. Cells were incubated with 10 μg/mL Hoechst‐33 342 and 0.25 μg/mL propidium iodide (PI) at 37 °C, 5% CO2 for 1 h before imaging. For the positive control, 99% ice‐cold ethanol was added simultaneously with the staining solution.

Images were acquired using the ImageXpress HT.ai (Molecular Devices) microscope at 10× magnification imaging six sites per well in 14 z‐steps (8 μm step size) through the DAPI channel and the TexasRed channel. Image analysis was performed on 2D‐projection images in the MetaXpress software (Molecular Devices, San Jose, CA, USA), quantifying the total number of cells (Hoechst positive nuclei) and the number of dead cells (Hoechst and PI positive nuclei) within organoids (minimum 40 μm diameter) using the Find Round Objects function.

### 2.9 Ethical considerations

The work and methodology used with clinical tumour samples has been carried out in accordance with Ethical principles for Medical Research of the World Medical Association Declaration of Helsinki. The experiments were undertaken with the understanding and written consent of each subject. The respective licence numbers for approval of the studies are as follows: licence no.: H‐21071553; OVACAN, from the Ethical committee of the Capital Region, Denmark, for the Danish cohort of HGSOC biopsies for preparation of patient‐derived organoids, collected at the University hospital, Copenhagen (the sample collection period is omitted for this cohort due to the Danish GDPR rules, as the samples from the three patients included in this study were collected in a short timeframe, possibly leading to potential disclosure of patients' identity); licence nos. H‐3‐2010‐095 & till. H‐3‐2011‐30 600 from the Ethical committee of the Capital Region, Denmark, for the Danish cohort of archival paraffin breast cancer samples for immunohistochemistry, collected at the Copenhagen University Hospital between August 2010 and June 2019; and licence no.: CEIM‐CHUIMI‐12/14/2017 from the Ethics Committee of the University Hospital of Las Palmas, Gran Canaria, for the archival paraffin samples of HGSOC for the immunohistochemistry study conducted in accordance with the Spanish Ley de Investigación Biomédica, collected at the Department of Pathology, University Hospital, Las Palmas, Gran Canaria, Spain between March 1995 and April 2005.

### 2.10 Kaplan–Meier plots

The kmplot website was used for the generation of the plots [28]. The mRNA gene chip databases were used. The best cut‐off was automatically selected [29]. For LUAD, 1161 patients with the adenocarcinoma histological subtype were selected. For TNBC, the ER status—immunohistochemically (IHC) defined, ER status–—array, PR status—IHC and HER2 status—array negative subtypes were selected, resulting in a total of 392 patients.

### 2.11 Statistical analysis

For the immunofluorescence staining results, each point represents the average mean intensity of a biological replicate, normalized to the respective mean intensity of the WT NT sample. Significance was calculated with the one‐way ANOVA test, using the GraphPad recommended correction for the multiple comparisons for Figs 3 and 6. For the ratio of the DNA‐cisplatin adduct in Figure S7, the calculated values came from the ratio: average of the mean intensity for the cisplatin‐treated sample/average of the mean intensity for the nontreated sample. Statistical significance was calculated with the unpaired *t*‐test. For the qPCR results, the bar represents the mean ± SD of three independent experiments. Significance was calculated with the one‐way ANOVA test, using the GraphPad recommended correction for multiple comparisons. For the tumour organoids experiments, the drug‐induced cell death is presented by normalizing to positive and negative controls, and IC50s were calculated from fit by nonlinear regression. Statistically significant differences between curve fits were evaluated by sum‐of‐squares F‐test. For 2D culture cytotoxicity assays, the normalized data were calculated in the ScanR Analysis software, and IC50s were calculated using the ‘[Inhibitor] vs. normalized response ‐‐ Variable slope Nonlinear regression’ function. Statistically significant differences between curve fits were evaluated by sum‐of‐squares F‐test. All the graphs and data analysis were generated in GraphPad Prism 10.2.3 or 10.6.0.

### 2.12 Antibodies and chemicals

These primary antibodies were used for immunofluorescence (IF) staining: anti‐Ubiquitin K48 (1 : 100, ZRB2150; Sigma‐Aldrich, St. Louis, Missouri, United States), anti‐phospho‐Histone H2A.X (1 : 500, 05‐636; Sigma‐Aldrich, USA), anti‐Cisplatin modified DNA (1 : 500, ab103261; Abcam, Cambridge, United Kingdom) and anti‐metallothionein (1 : 500, NBP1‐97493; Novus Biologicals). Primary and secondary antibodies used for immunoperoxidase cyto‐ and histo‐chemistry are specified in section 2.6. The secondary antibodies used for the IF staining were as follows: goat anti‐Mouse IgG Alexa Fluor™ 647 (1 : 1000, A‐21236; Invitrogen, Waltham, Massachusetts, USA), goat anti‐Rabbit IgG Alexa Fluor™ 750 (1 : 1000, A‐21039; Invitrogen, USA) and goat anti‐Rat IgG Alexa Fluor™ 647 (1 : 1000, A‐21247; Invitrogen, USA). These primary and secondary antibodies used for western blotting were as follwows: anti‐MTF1 (1 : 1000, 25 383‐1‐AP; Proteintech, Rosemont, IL, USA), beta‐actin (1 : 5000, A1978; Sigma‐Aldrich, USA), Goat Anti‐Rabbit IgG (H + L) Peroxidase (1 : 10000, PI‐1000‐1; Vector Laboratories, Newark, CA, USA) and Horse Anti‐Mouse IgG (H + L) Peroxidase (1 : 10000, PI‐2000; Vector Laboratories). The following chemicals and drugs were used: CuET (D0487; TCI Chemicals, Tokyo, Japan), prepared as a 2.5 mm stock solution in dimethyl sulfoxide (DMSO, 1029311000; Sigma‐Aldrich, USA); cisplatin (598 049; Accord Healthcare B.V. via Nomeco, Copenhagen, Denmark); and carboplatin (439 635; Accord Healthcare B.V. via Nomeco, Copenhagen, Denmark).

## 3 Results

### 3.1 Establishment and validation of a panel of cisplatin‐resistant cancer cell lines

As the first step towards the goals of this study, we sought to establish a panel of matched pairs of human carcinoma cell line models sensitive and resistant to cisplatin treatment, respectively. We selected four commonly used lung adenocarcinoma (LUAD) cell lines: H1792, HCC4006, A549 and H1944, and two widely studied triple‐negative breast cancer (TNBC) cell lines: MDA‐MB‐231 and MDA‐MB‐468. First, we confirmed by repeated cytotoxicity assays that these six parental cell lines, referred to as wild‐type (WT), were all sensitive to micromolar concentrations of cisplatin. Next, all the six WT cell lines were subjected to long‐term cell culture conditions under increasing concentrations of cisplatin (Fig. 1A), to derive syngeneic paired cell lines resistant to cisplatin (referred to as ‘cisR’). The comparative cytotoxicity assay was then used to estimate IC_50_ values for cisplatin, for both the WT and cisR cell lines (Fig. 1B, C). To better document the cytotoxicity results, the cell survival curves for each cell line pair under treatment with increasing concentrations of cisplatin are presented in Fig. 1D.

**Fig. 1:**
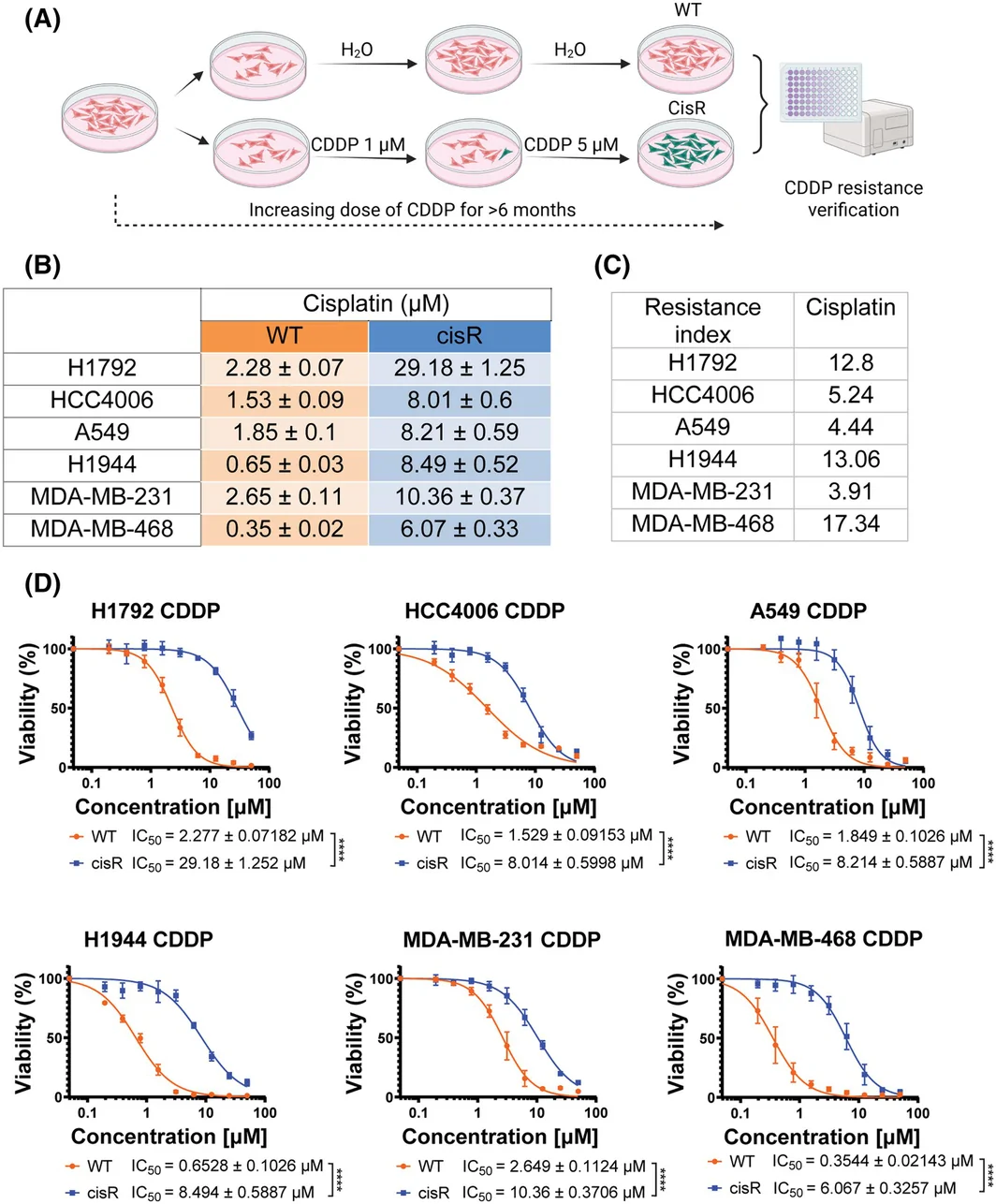
Cisplatin‐resistant (cisR) lung adenocarcinoma (LUAD) and triple‐negative breast cancer (TNBC) cell lines were established and validated by cisplatin treatment. (A) Scheme to prepare the cisplatin‐resistant human cancer cell lines, each selected as uncloned bulk cell population emerging under long‐term selection of the parental cell line in increasing doses of cisplatin. (B) Table comparing IC50s of parental cells and their cisplatin‐resistant derivatives. (C) The resistance indexes for cisplatin for each cell line. Values were calculated as the ratio of the cisR IC_50_/WT IC_50_ for each cell line. (D) The cell line survival curves for the cytotoxicity assay from which IC50s were calculated. Cells were plated in 96‐well plates. Forty‐eight hours later, they were treated with a range of cisplatin (CDDP, 0–50 μμ) concentrations. Following 96 h of incubation, the plates were stained with Hoechst and SYTOX™ Deep Red analysed in a scanR High‐Content Screening Station. *n* = 3. Statistically significant differences between curve fits were evaluated by sum‐of‐squares *F*‐test: ****P < 0.0001, ***P < 0.001, **P < 0.01, *P < 0.05, ns, nonsignificant, IC50 ± SE.

### 3.2 CuET is cytotoxic for both cisplatin‐sensitive and cisplatin‐resistant cancer cell lines

Next, we employed the same cell cytotoxicity assay and assessed the responses of the six matched cell line pairs to CuET, to (i) define the sensitivities of the parental WT cell lines; and (ii) examine whether the acquired resistance to cisplatin is associated or not with gained cross‐resistance against CuET. As can be seen from the IC_50_ values for treatment with CuET, all six WT cell lines were sensitive to CuET (Fig. 2A). Notably, the IC_50_ values for treatment with CuET remained virtually unchanged when the matched pairs of WT and cisR cell lines were compared side by side, as also indicated by the resistance index (RI), calculated as the IC_50_ ratios of cisR/WT cell lines, which were in all cases below 2.0, a value regarded as the lowest threshold (21) to consider resistance (Fig. 2A–C). This preserved sensitivity to CuET among the cisR models contrasted sharply with generally much higher RIs for cisplatin. Thus, among the LUAD models the lowest RI for cisplatin treatment was 4.44 (A549), followed by HCC4006 with RI of 5.24. The highest RIs were 12.8 and 13.06 for H1792 and H1944, respectively (Figs 1C and 2B). In the TNBC models, the RI value for cisplatin toxicity was 3.91 for MDA‐MB‐231 and 17.34 for MDA‐MB‐468 cells, respectively. Indeed, unlike the robust RIs for cisplatin treatment, the highest RI for CuET was 1.78 (HCC4006) among our LUAD models and 1.09 (MDA‐MB‐231) in the TNBC models (Fig. 2B).

**Fig. 2:**
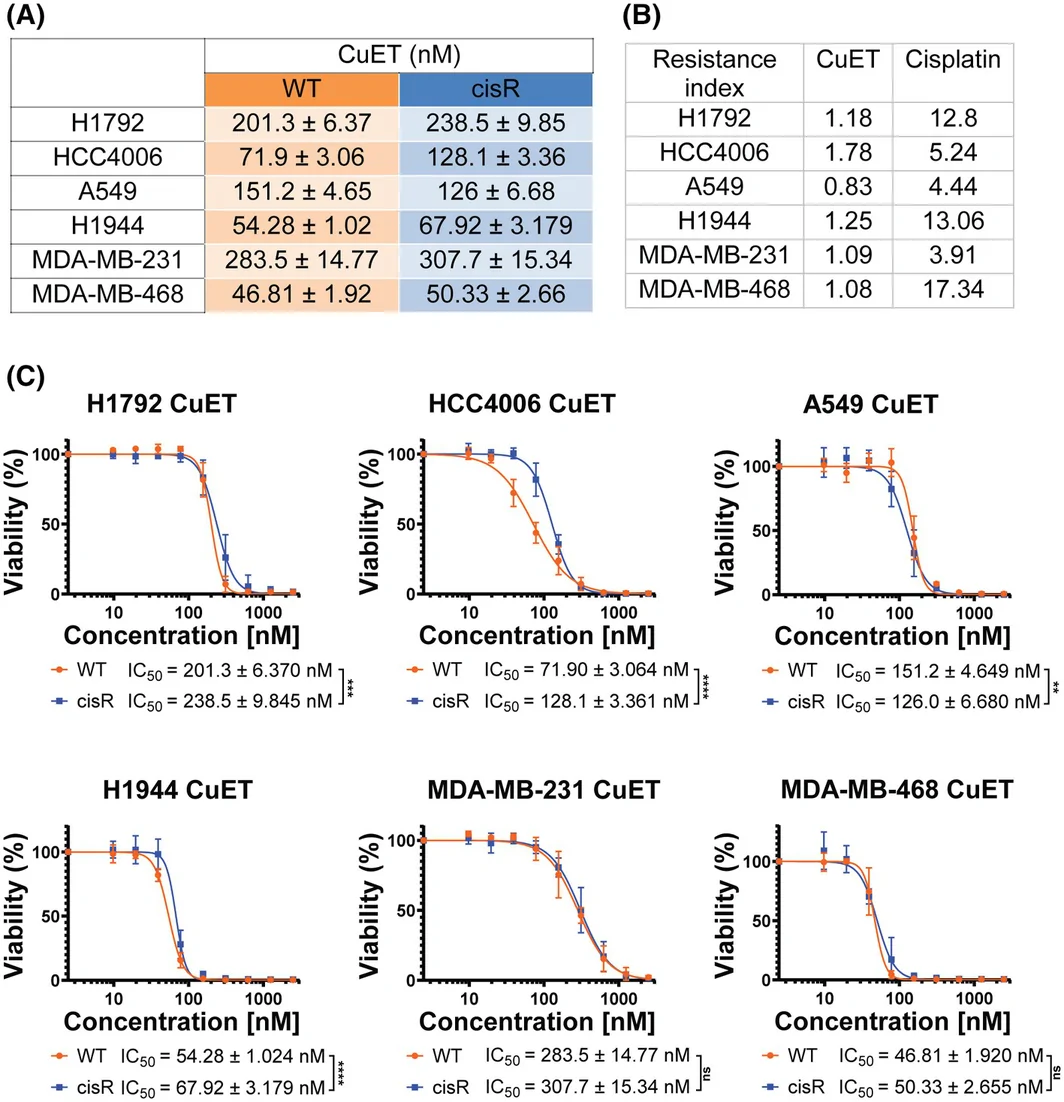
Parental wild‐type (WT) and cisplatin‐resistant (cisR) lung adenocarcinoma (LUAD) and triple‐negative breast cancer (TNBC) cell lines are similarly sensitive to CuET. (A) Table showing IC50s derived from survival assays. (B) The resistance indexes for CuET and cisplatin for each cell line. Values were calculated as the ratio of the cisR IC_50_/WT IC_50_ for each cell line. (C) The cell line survival curves for the cytotoxicity assay from which IC50s were calculated. Cells were plated in 96‐well plates. Forty‐eight hours later, they were treated with a range of CuET (0–2500 nm) concentrations. Following 96 h of incubation, the plates were stained with Hoechst and SYTOX™ Deep Red analysed in a scanR High‐Content Screening Station. *n* = 3. Statistically significant differences between curve fits were evaluated by sum‐of‐squares *F*‐test: ****P < 0.0001, ***P < 0.001, **P < 0.01, *P < 0.05, ns = nonsignificant, IC50 ± SE.

Together, these results indicate that acquired resistance to cisplatin in our carcinoma cell line models does not confer cross‐resistance to CuET.

### 3.3 Proteotoxic vs. genotoxic drug effects, and diverse causes of cisplatin resistance

To assess the likely major causes of cytotoxicity for CuET vs. cisplatin, and to gain insights into the acquired cisplatin resistance in our models, we next assessed functional readouts for proteotoxic: accumulation of K48‐(poly)ubiquitinated proteins and genotoxic stress: platinum DNA adducts and phosphorylated histone H2AX (γH2AX), respectively, in all 12 cell lines.

First, as examined by quantitative IF in Triton X‐100‐pre‐extracted cells, all cell lines showed enhanced accumulation of K48‐ubiquitinated proteins upon a 4‐h exposure to CuET (Fig. 3A; Fig. S1). Furthermore, treatment with cisplatin did not evoke any significant changes in the accumulation of K48‐tagged proteins in any of the cell lines (Fig. 3A; Fig. S1), identifying this parameter [12] as a phenotypic effect selective for CuET. Examples of original data are shown in Figs S2 and S3.

**Fig. 3:**
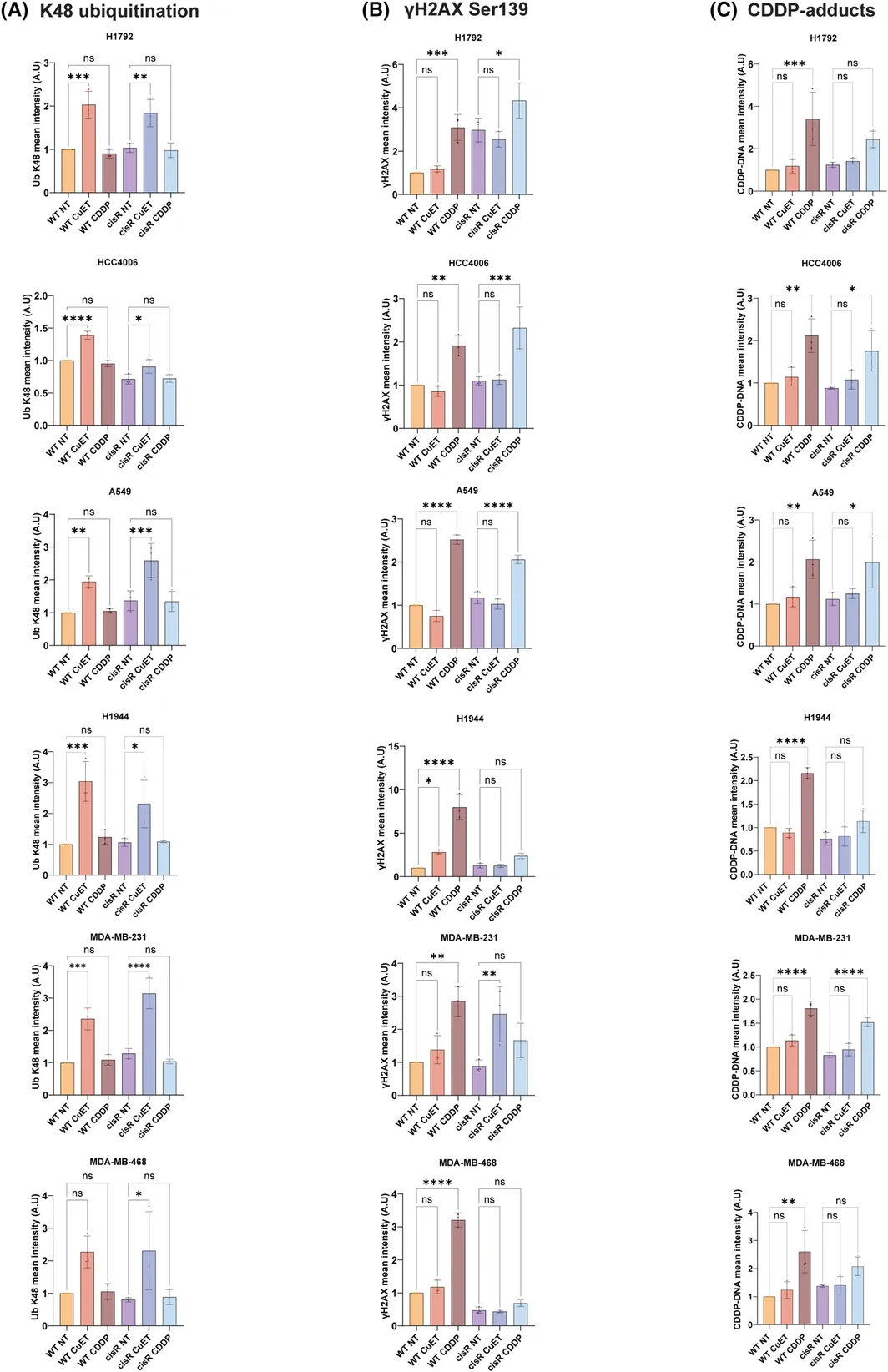
CuET induces accumulation of K48 ubiquitinated cellular proteins, while cisplatin induces DNA damage response and formation of DNA adducts in wild‐type (WT) and cisplatin‐resistant (cisR) cell lines. (A) Proteotoxic stress was assessed via K48 ubiquitinated proteins staining in pre‐extracted samples. (B) DNA damage was assessed via γH2AX Ser139 staining. (C) Cisplatin internalization was evaluated via CDDP‐adducts staining. Cells were treated with 1μm CuET and 20 μm cisplatin (CDDP) for 4 h. Samples were analysed in a scanR High‐Content Screening Station. Statistical significance was assessed with the one‐way ANOVA test. ****P < 0.0001, ***P < 0.001, **P < 0.01, *P < 0.05, ns, nonsignificant, NT, nontreated. Data shown as mean with SD, *n* = 3.

To further assess the responses of our models to both cisplatin and CuET, evaluation of the drug‐induced DNA damage was performed by IF analysis of γH2AX levels [28] as a general marker of DNA damage response (Fig. 3B, Figs S4–S6). In parallel, formation of cisplatin‐DNA adducts was examined by IF analysis with an antibody recognizing cisplatin modified DNA (Fig. 3C, Figs S7–S9) [29]. While all six parental (WT) cell lines showed increased γH2AX intensity after 4‐h cisplatin treatment (Fig. 3B), this parameter was significantly lower in four of the six cisR cell models compared with their respective WT counterparts (Fig. S4), despite all cell lines being exposed to identical doses of cisplatin for identical times. Notably, the cisRH1944 cells displayed significantly lower cisplatin adducts, while the other cisR cell lines showed either the same or only partially reduced adduct levels compared with their WT matched lines (Fig. 3C, Figs S7–S9). Thus, our cisR models can be subclassified according to their patterns of DNA damage markers, into those with: (i) preserved both γH2AX and cisplatin adduct levels (HCC4006 and MDA‐MB‐231); (ii) reduced both γH2AX and cisplatin adducts (H1944); and (iii) reduced γH2AX despite preserved or only moderately reduced cisplatin adducts (H1792, A549 and MDA‐MB‐468) (Figs S4 and S7).

Interestingly, CuET induced DNA damage in two of the six cell lines: H1944 WT and MDA‐MB‐231 cisR cells (Fig. 3B), highlighting the context‐dependent effects the drug can display, in addition to the generally observed proteotoxic stress.

Altogether, these data are consistent with distinct cytotoxicity mechanisms evoked by CuET (mainly proteotoxic stress) versus cisplatin (DNA damage), indicate context‐dependent genotoxic effects of CuET in some models, and suggest diverse mechanisms that may underlie the resulting cisplatin resistance.

### 3.4 CuET is highly cytotoxic for ovarian cancer models

As platinum‐based drugs are also used to treat ovarian cancer, and HGSOC is a therapeutic challenge due to commonly occurring platinum resistance [30, 31, 32, 33], we next examined CuET effects on two types of human ovarian cancer models. First, we compared CuET with the standard‐of‐care drugs cisplatin and carboplatin in an ovarian cancer cell line OVCAR‐5 (Fig. 4A–D). While both cisplatin and carboplatin were cytotoxic, there was a fraction of cells that survived the treatment even at high drug concentrations (Fig. 4A,C,D). In contrast, CuET reduced the surviving OVCAR‐5 population close to zero at low drug concentrations (Fig. 4B,C). Mechanistically, CuET, but neither cisplatin nor carboplatin, evoked proteotoxic stress, evidenced as increased K48‐ubiquitinated proteins (Fig. 4E,F). DNA damage (γH2AX) was induced upon treatment with CuET and cisplatin, but not carboplatin (Fig. 4G,H).

**Fig. 4:**
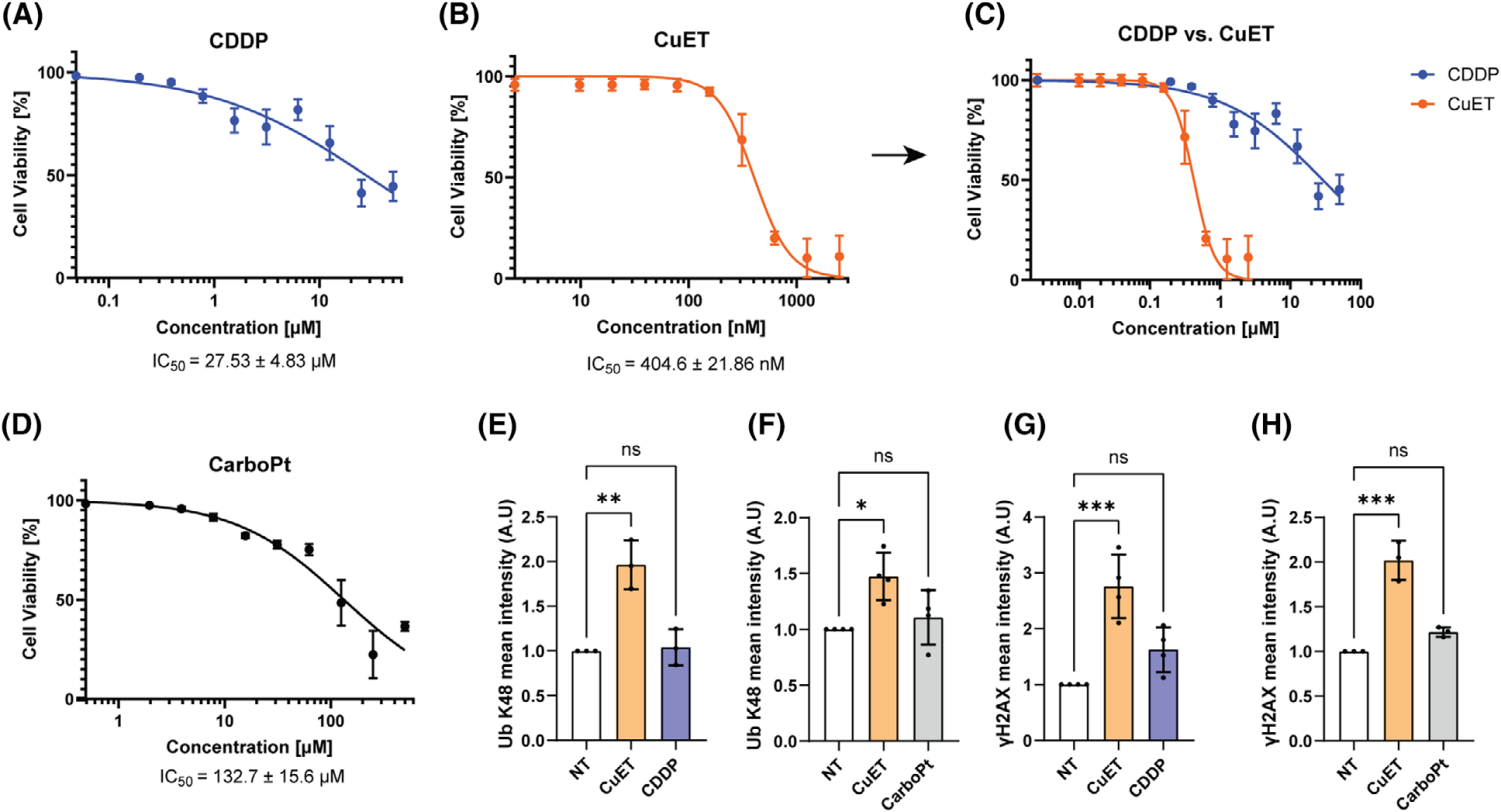
Ovarian cancer 2D models are highly sensitive to CuET compared with cisplatin and carboplatin, as shown by cytotoxicity assays and immunofluorescence (IF) analysis of K48 ubiquitinated proteins and γH2AX Ser139 levels. Survival curves for cytotoxicity assay of OVCAR‐5 treated by (A) cisplatin, (B) CuET and (D) carboplatin. (C) Combined graph of curves from (A) and (B) comparing cisplatin and CuET treatment. Cells were plated in 96‐well plates. Forty‐eight hours later, they were treated with a range of cisplatin (0–50 μm), CuET (0–2500 nm) and carboplatin (0–500 μm) concentrations. Following 96 h of incubation, the plates were stained with Hoechst and SYTOX™ Deep Red analysed in a scanR High‐Content Screening Station. *n* = 3. IC50 ± SE was calculated as shown under the graphs. (E–H) Proteotoxic stress was assessed via K48 ubiquitinated proteins staining, and DNA damage was assessed via γH2AX Ser139 staining. Cells were treated with 1μm CuET, 20 μm cisplatin (CDDP) and 200 μm carboplatin for 4 h. Samples were analysed in a scanR High‐Content Screening Station. Statistical significance was assessed with the one‐way ANOVA test. ****P < 0.0001, ***P < 0.001, **P < 0.01, *P < 0.05, ns, nonsignificant, NT, nontreated. Data shown as mean with SD, *n* = 4 (E, H), *n* = 3 (F, G).

Second, we established a series of 3D organoids from HGSOC biopsies taken before chemotherapy onset (see the Methods section). Three of these PDO models, from patients who developed early recurrence due to platinum resistance of their tumours, were then subjected to treatments with CuET, cisplatin and carboplatin (Fig. 5A–E). The results were overall consistent with those obtained with the OVCAR‐5 cells, in that all three organoid models were more sensitive to CuET than to cisplatin and carboplatin, the trend again reflected also by the lowest fraction of surviving tumour cells after treatment with CuET (Fig. 5A–E).

**Fig. 5:**
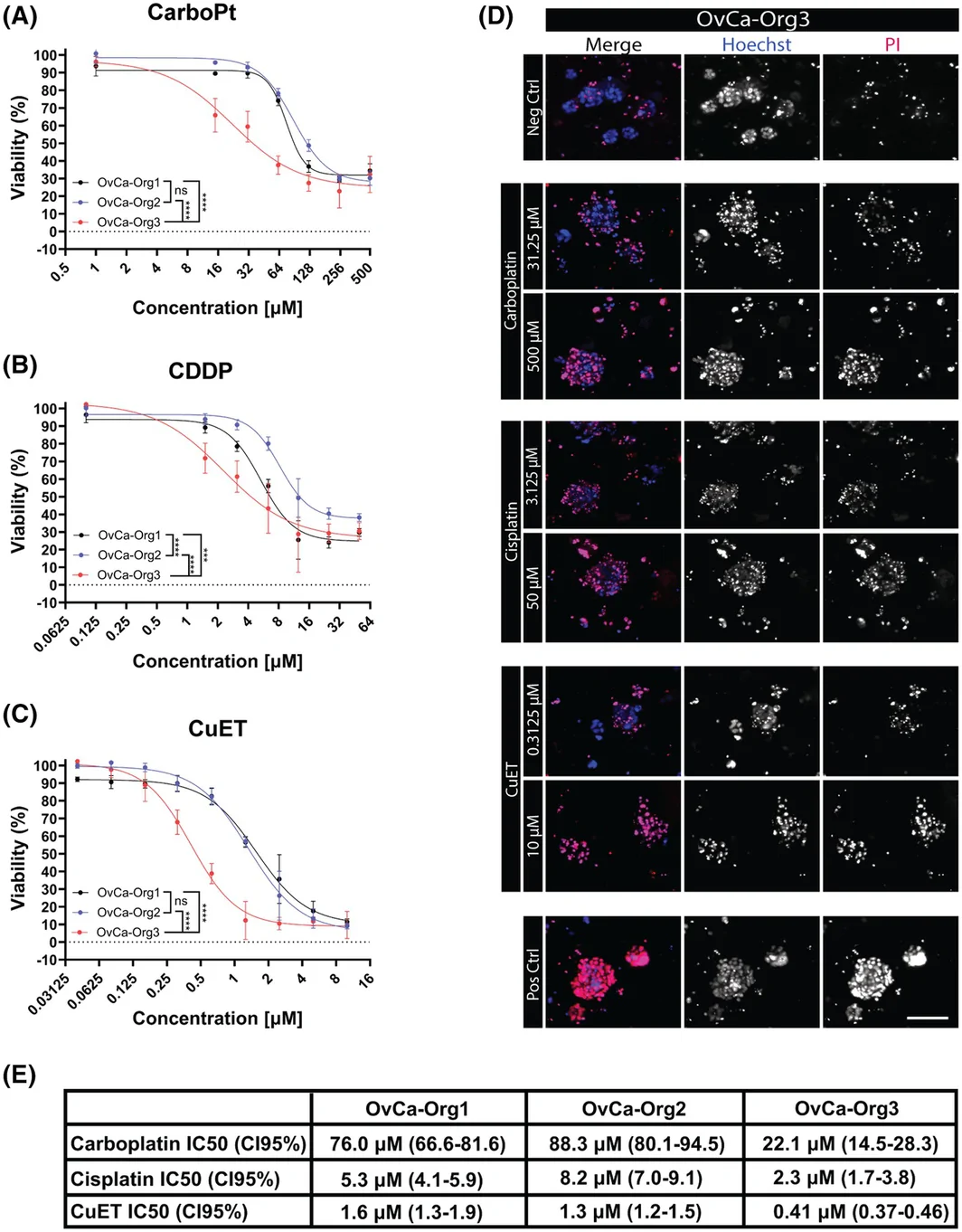
High‐grade serous ovarian carcinoma (HGSOC) organoids are highly sensitive to CuET in comparison with platinum drugs. Organoids of HGSOC tumours (OvCa‐Org) from three different patients were treated with twofold increasing concentrations of (A) carboplatin (CarboPt), (B) cisplatin (CDDP) and (C) CuET for 96 h before evaluating remaining viable cells as percentage of total cell count using Hoechst and propidium iodide (PI) staining (data shown on Log2 scale as mean ± SD of two biological replicates with duplicate samples). Representative images are shown in (D). Images were acquired at 10X magnification (scale bar, 100 μm) with ImageXpress confocal HT microscopy. Curves were fitted by nonlinear regression and IC50 values were calculated from this, which are shown in (E). Statistically significant differences between curve fits were evaluated by sum‐of‐squares *F*‐test: ****P < 0.0001, ***P < 0.001, **P < 0.01, *P < 0.05, ns, nonsignificant, NT, nontreated.

These data extended the validation of CuET as a potentially valuable compound to treat also ovarian carcinomas, as indicated by the more complete eradication of cancer cells and at clinically achievable drug concentrations, surpassing the effects of the standard‐of‐care platinum drugs.

### 3.5 Metallothionein expression levels respond to CuET, and mitigate CuET's toxicity

Inspired by the ability of CuET to overcome cisplatin resistance, and the fact that in a high‐throughput screen disulfiram was most toxic for cancer cell lines with low levels of metallothioneins [19, 34], a family of metal‐detoxification proteins that have also been implicated in counteracting toxicity of diverse drugs [35], we next assessed metallothioneins as potential mitigators of toxicity evoked by the copper‐containing CuET. We first focused on two members of the metallothionein family, *MT2A* and *MT1E*, known to be involved in the detoxification of both heavy metals, such as cadmium in tobacco smoke [36], and chemotherapeutics, including cisplatin [37, 38, 39]. Examination of mRNA levels for *MT2A* (Fig. 6A, Fig. S10A) and *MT1E* (Fig. 6B, Fig. S10B) by quantitative polymerase chain reaction (qPCR), in our six pairs of matched WT and cisR cell lines treated or not with CuET or cisplatin revealed the following: (i) The mRNAs of the two *MT* genes showed moderately different levels among the diverse cell lines under basal untreated conditions; (ii) Treatment with CuET evoked upregulation of one or both *MT* genes across all cell lines, with the exception of H1944 (WT/cisR) and WT A549 cells where the trend towards increased expression of *MT‐2A* did not reach statistical significance; and (iii) In contrast to CuET, cisplatin treatment did not show any profound effect on the mRNA expression of either of the two metallothionein genes in any of the 12 cell lines. Next, we wished to assess metallothionein protein abundance and subcellular expression patterns, also given the fact that CuET treatment negatively impacts protein synthesis [16]. While our attempts to apply western blotting to detect endogenous MTs with an antibody raised against MT1 and MT2 were unsuccessful, our immunofluorescence analysis showed different responses of MT patterns to cisplatin vs. CuET (Fig. 6C). Thus, unlike in control or cisplatin‐treated MDA‐MB‐231 cells, CuET exposure evoked enhanced accumulation of immobilized, nonextractable MT proteins (Fig. 6C), an observation that might reflect direct binding of MTs to the copper‐containing CuET, thereby potentially ‘neutralizing’ the intracellular CuET and reducing its cytotoxicity.

**Fig. 6:**
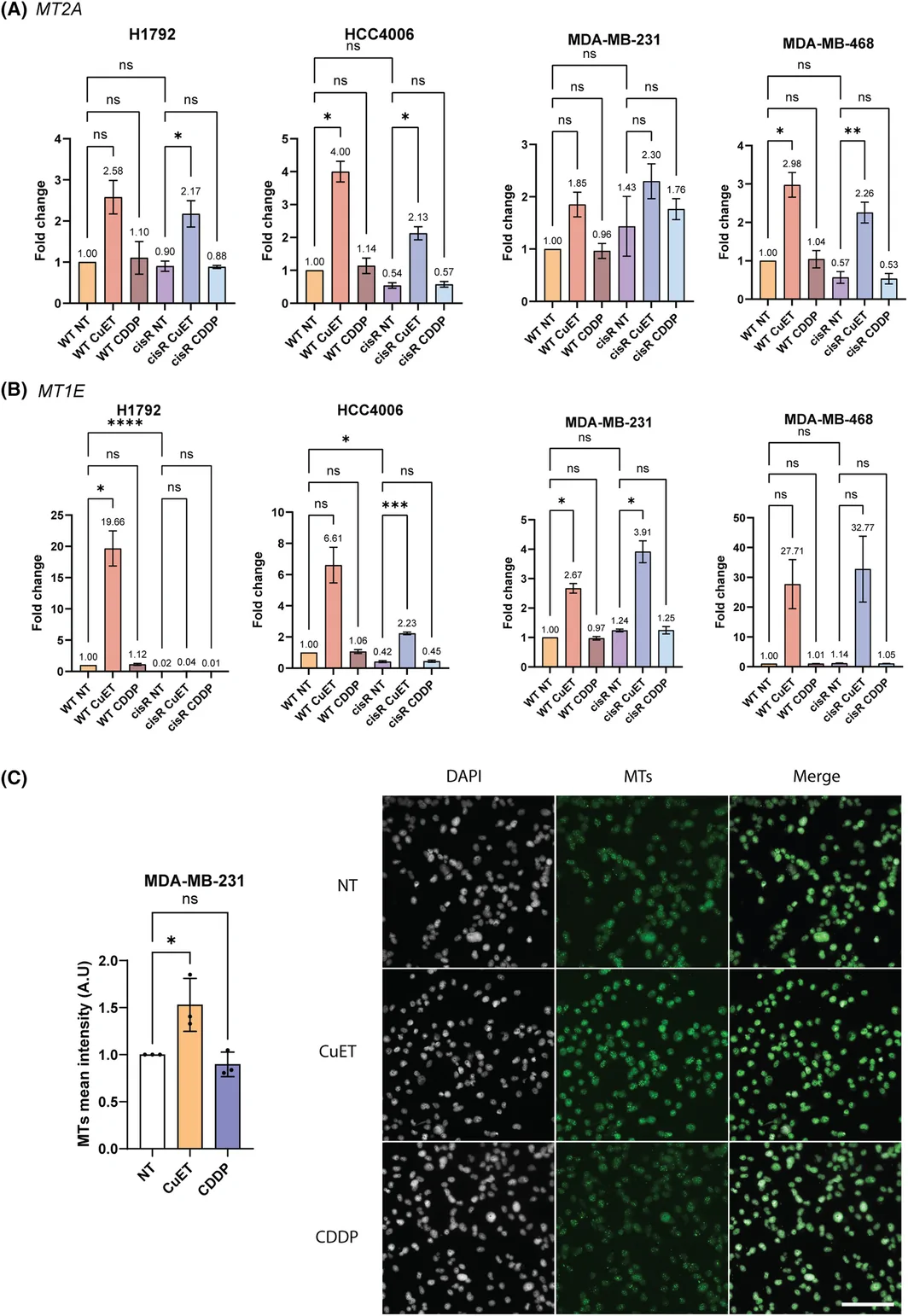
CuET, but not cisplatin exposure alters metallothionein expression patterns in representative cell lines. The mRNA expression levels of (A) *MT2A* and (B) *MT1E* were evaluated via qPCR. Cells were treated with 1 μm CuET and 20 μm cisplatin (CDDP) for 4 h. (C) The metallothionein protein pattern was evaluated via QIBC in MDA‐MB‐231 cells. Cells were treated with 1 μm CuET and 20 μm cisplatin (CDDP) for 6 h. Representative images and summary graph from three independent immunofluorescence staining experiments are shown. Statistical significance in (A–C) was assessed with the one‐way ANOVA test. ****P < 0.0001, ***P < 0.001, **P < 0.01, *P < 0.05, ns, nonsignificant, NT, nontreated, scale bar = 100 μm, Data shown as mean with SD, *n* = 3.

Next, to test whether the metallothionein proteins might modulate the CuET toxicity in a subset of our models, we knocked down Metal Transcriptional Factor 1 (MTF1), the key transcription factor known to regulate metallothionein gene expression [40]. MTF1 is itself an inducible transcription factor that responds to conditions, such as heavy metal exposure, hypoxia, oxidative stress and hyperthermia [40]. Using two different siRNAs, we successfully reduced the MTF1 protein in three independent experiments (Fig. 7A, Fig. S11A). Indeed, MTF1 depletion was accompanied by enhanced cytotoxicity of CuET in both cell lines tested (Fig. 7B,C). In another series of independent experiments, using the more potent of the two siRNAs (tested in Fig. 7A–C) and a complementary approach as further validation of the cytotoxicity results, siRNA‐mediated reduction of *MTF1* led to a significant decrease in IC_50_ values for the three model cell lines treated by CuET (Fig. 7D, Fig. S11B). Parallel control immunocytochemistry experiments confirmed that the *MTF1* knock‐down indeed resulted in decreased levels of MT proteins as detected by staining with antibody to MT1 and MT2 (Fig. S11C) and detailed measurements of mRNA confirmed the impact of the partial knock‐down on mRNA levels of *MTF1* itself, as well as its *MT* transcriptional targets, particularly *MT1E*, in all three cell lines tested (Fig. 7E, Fig. S11D). Altogether, these results indicated that metallothionein levels may influence the cancer cell response to CuET in our lung and breast cancer models.

**Fig. 7:**
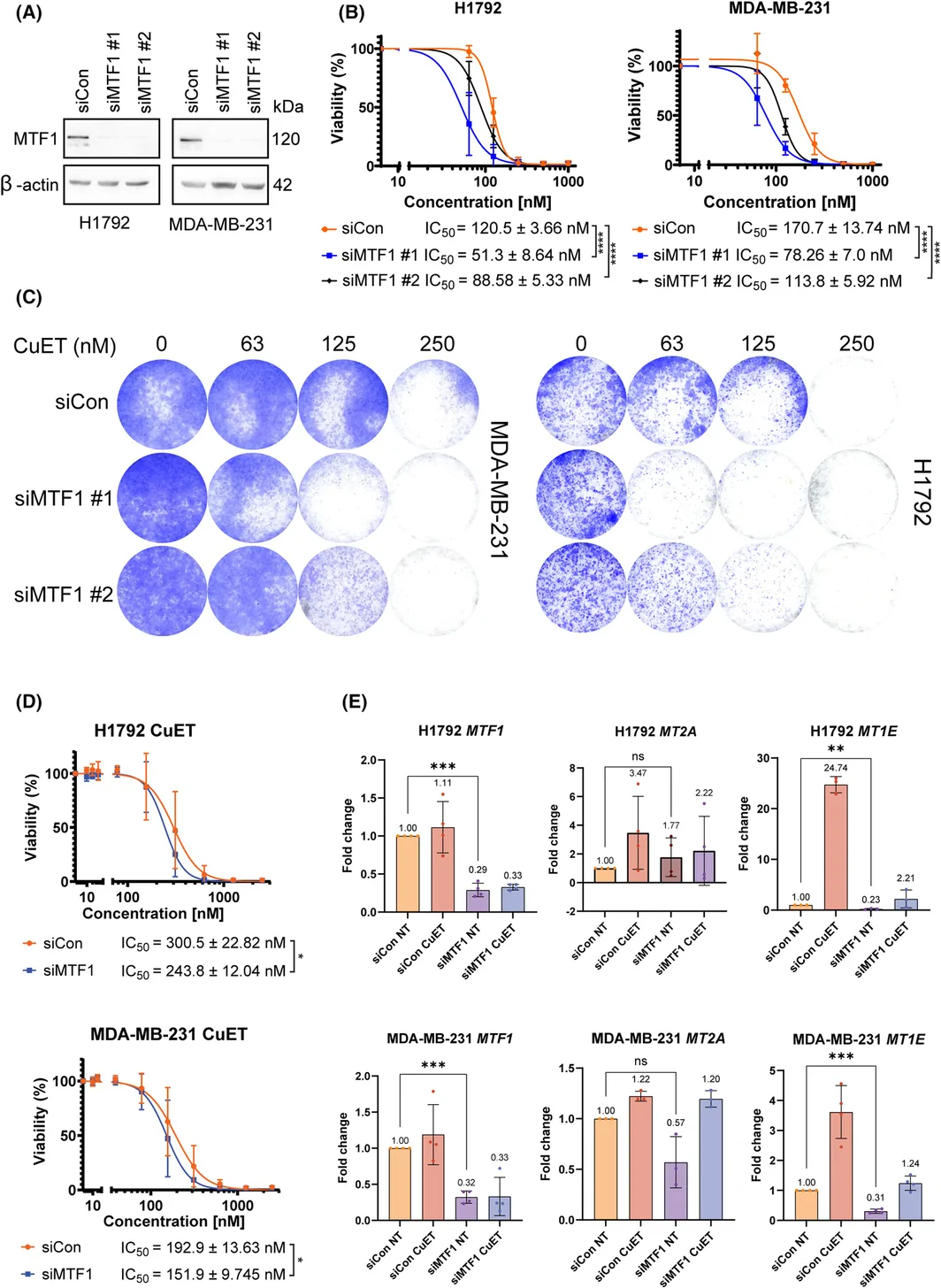
Knock‐down of MTF1 leads to lower *MT1‐E* and *MT2A* expression and partly enhances the cytotoxic effect of CuET in H1792 wild‐type (WT) and MDA‐MB‐231 WT cells. (A) Western blotting verification of MTF1 depletion in the indicated cell lines, using two different siRNAs (siRNA #1 Ambion, siRNA #2 OriGene. *n* = 3). (B) Impact of MTF1 knock‐down (from A) on CuET cytotoxicity (crystal violet assay, see the Methods section) in the indicated cell lines treated with a range of CuET (0–1000 nm) concentrations. *n* = 3. Statistically significant differences between curve fits were evaluated by sum‐of‐squares *F*‐test: ****P < 0.0001, ***P < 0.001, **P < 0.01, *P < 0.05, ns, nonsignificant, IC50 ± SE. (C) Representative images of crystal violet staining from assay shown in 7B. (D) Alternative cytotoxicity assays: 24 h after knock‐down (siMTF1 #1), H1792 WT and MDA‐MB‐231 WT cells were plated in 96‐well plates. Forty‐eight hours later, they were treated with a range of CuET (0–2500 nm) concentrations. Following 96 h of incubation, the plates were stained with Hoechst and SYTOX™ Deep Red analysed in a scanR High‐Content Screening Station. *n* = 4. Statistically significant differences between curve fits were evaluated by sum‐of‐squares *F*‐test: ****P < 0.0001, ***P < 0.001, **P < 0.01, *P < 0.05, ns, nonsignificant, IC50 ± SE. (E) 24 h after knock‐down (siMTF1 #1), H1792 WT and MDA‐MB‐231 WT cells were plated in 6‐well plates. Forty‐eight hours later, they were treated with 1μm CuET. Following 4 h of incubation the expression levels of *MTF1, MT2A* and *MT1E* were evaluated via qPCR. Statistical significance was assessed with the one‐way ANOVA test. ****P < 0.0001, ***P < 0.001, **P < 0.01, *P < 0.05, ns, nonsignificant, NT, nontreated. Data shown as mean with SD, *n* = 3.

### 3.6 Metallothioneins as candidate biomarkers for future CuET‐based therapy

To assess the likelihood of their potential as candidate predictive biomarkers for any future CuET‐based therapy, we then employed immunohistochemistry to examine expression patterns of metallothioneins and NPL4 (the CuET target protein whose ectopic overexpression caused resistance to CuET in cell culture models, [12]) in specimens from previously studied clinical cohorts [20, 21, 22, 23, 24] of triple‐negative breast cancer (TNBC) and HGSOC.

We classified the staining patterns of the ubiquitous NPL4 protein as either normal or high (see Methods section 2.6), the latter predicted to potentially cause resistance to CuET, based on our mechanistic study of the NPL4‐CuET interplay [12]. As can be seen from the staining examples and summary graphs in Fig. 8A,C, the vast majority of TNBC tumours showed ubiquitously expressed NPL4, at levels closely matching those in normal breast epithelium. Among the HGSOC tumours, again the majority of cases showed moderate levels of NPL4, while 8% of cases displayed high NPL4 (Fig. 8B,C).

**Fig. 8:**
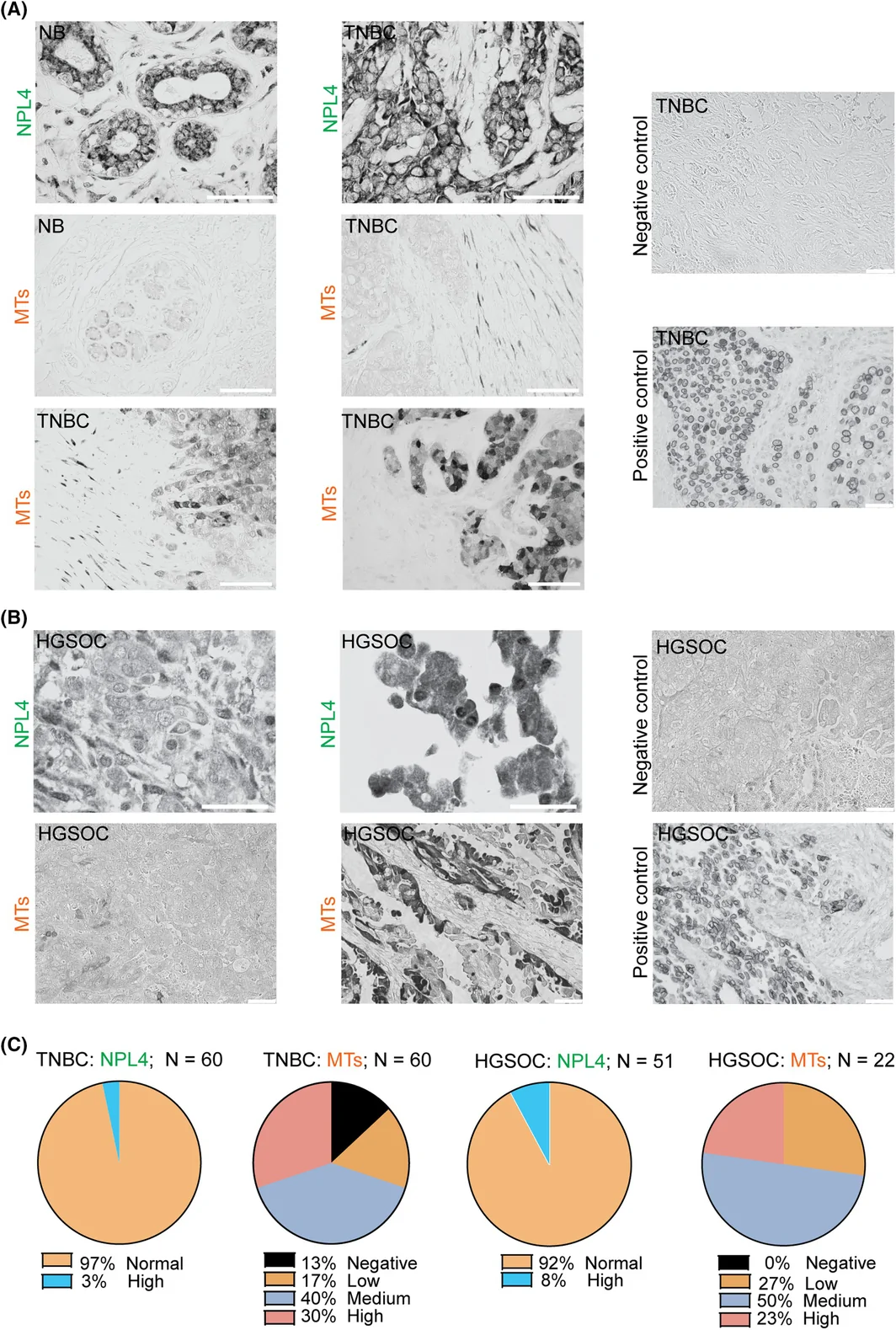
Metallothionein but not NPL4 expression varies in clinical samples of triple‐negative breast cancer (TNBC) and high‐grade serous ovarian carcinoma (HGSOC) as shown by immunohistochemistry (IHC). Representative examples of immunohistochemical staining patterns of NPL4 and metallothioneins (MTs), the latter with antibody to MT1 and MT2 (see the Methods section). In (A) normal breast (NB), TNBC and (B) ovarian cancer (HGSOC) tissues. Scale bar = 50 μm. (C) Pie charts visualizing fractions of patient samples stained for NPL4 and MTs; immunohistochemistry method, scoring categories, negative and positive (nucleoporin TPR) controls are explained in the Methods section.

For metallothioneins, we classified the staining patterns into four categories based on the fraction of positive cells (see the Methods section), as unlike for NPL4, both low/negative and high expression levels might be expected, with potentially differential impact on CuET sensitivity. The representative images of staining with the metallothionein antibody raised against MT1 and MT2, and the summary graphs for the TNBC tumours are shown in Fig. 8A,C, and for the HGSOC in Fig. 8B,C. Overall, the heterogeneity of metallothionein was much more pronounced, with approximately 30% of cases for both TNBC and HGSOC showing negative/low expression, and some 20–30% of highly positive tumours, with the remaining fraction showing an intermediate pattern.

While the clinical relevance of these results is considered in the Discussion section, the much higher degree of heterogeneity suggests that metallothioneins, rather than NPL4, might warrant evaluation as a potential predictive biomarker to help guide future clinical trials with CuET. Relevant to this notion, Kaplan–Meier survival plots obtained through datamining the kmplot website [41, 42], revealed that *MT2A* and *MT1E* levels correlate inversely with the overall survival of patients with LUAD and TNBC (Fig. S12).

Taken together, metallothionein protein expression patterns showed pronounced variability among the TNBC and HGSOC samples, as well as high heterogeneity within the individual lesions. In contrast, NPL4 was expressed rather uniformly across the TNBC and HGSOC cohorts, and at levels in the vast majority of tumours close to those in normal tissues. The biological significance and potential clinical implications of these results are discussed below.

## 4 Discussion

We believe that several aspects of our present study can significantly contribute to better understanding and overcoming platinum resistance in cancer treatment, a major challenge that even the second‐ and third‐generation platinum drugs have not been able to fully resolve [43].

First, the panel of our new human platinum‐resistant carcinoma models reported here can serve as a resource for scientists working in this research area. As establishment and validation of such models is time‐ and labour‐consuming, our models of carcinomas of the lung, breast and ovary are likely to be useful for example in screening efforts to identify new compounds or approved drugs that might overcome platinum resistance, or for more detailed studies of the molecular mechanisms underlying such chemoresistance. The latter application is already suggested by our present data on the platinum DNA adducts and γH2AX in response to cisplatin. Thus, in those cisR cell lines where a standard dose of cisplatin evoked lower degree of the adducts, cisplatin resistance might reflect aberrantly enhanced efflux or more efficient metabolic detoxification of the drug, for example. On the contrary, in the cisR models with adduct levels similar to the parental WT cells yet showing lower DNA damage signalling (γH2AX) after cisplatin treatment, the resistance might be due to enhanced DNA repair of the lesions, defective detection or signalling of the adducts, for example, while the cisR model with both the adduct and γH2AX preserved at levels similar to those in the parental cells, may be defective, for example, in checkpoint effector pathways, including cell death mechanism(s).

Second, and arguably the most significant result, is our finding that CuET is cytotoxic to cancer cells regardless of whether they are sensitive or resistant to cisplatin, suggesting CuET warrants further evaluation as a potential compound to treat cancer patients whose tumours do not respond well to standard‐of‐care platinum drugs. Furthermore, as the mechanisms of action for platinum drugs and CuET are distinct and, as we report here, they apparently do not share cross‐resistance, a combinatorial treatment could also be beneficial, a notion supported by findings from a phase II clinical trial with lung cancer where disulfiram addition to cisplatin and vinorelbine chemotherapy significantly improved patients' overall survival [44]. Another example of CuET's emerging value in overcoming tumour chemoresistance is multiple myeloma, where preclinical and *ex vivo* analyses of tumour biopsies including from patients with myelomas resistant to standard‐of‐care drugs bortezomib and carfilzomib showed sensitivity to CuET [15]. Also consistent with this notion are the results of a large epidemiological study from Denmark, where disulfiram use by cancer patients was associated with favourable prognosis across many cancer types, including metastatic stages, which are commonly resistant to standard chemotherapy [12].

Third: Both the emerging preclinical data (including those in our present study) as well as parallel efforts in several countries to develop clinically applicable forms of CuET suggest that CuET may soon be tested in clinical trials. This prospect raises a need for identification and validation of predictive biomarkers that might help patient selection for such clinical testing. Our present assessments of immunohistochemical patterns for NPL4 and metallothioneins in clinical cohorts of TNBC and HGSOC provide useful information relevant to this issue. While NPL4 caused resistance when overexpressed in cell culture models [12], our present results show that it is rarely if ever robustly elevated in clinical settings and therefore unlikely to serve as a clinically useful biomarker. On the contrary, the much more variable patterns of metallothioneins observed in our present analyses of both TNBC and HGSOC appear to be more encouraging in this respect. Based on our present results, and considering the known ability of metallothioneins to counteract toxic effects of drugs and heavy metals [35, 36], we propose that metallothioneins warrant further evaluation as candidate predictive biomarkers of responses to the copper‐containing CuET. While our present preclinical data are promising, the therapeutic potential of CuET in platinum‐resistant disease will ultimately require formal *in vivo* validation and clinical testing.

Fourth: At the conceptual level relevant to drug repurposing in general, our example of CuET as the active anticancer metabolite of disulfiram (DSF) highlights the significance of focusing on metabolites of clinically approved drugs, in addition to or instead of the (pro)drugs themselves. As a metabolite of the alcohol‐abuse drug DSF, CuET has a decades‐long history of safety and tolerability in humans, and its anticancer properties [12, 13, 14, 15] provide a reasonable prospect of a relatively inexpensive, rapid and potentially successful clinical applications in oncology. In addition, CuET avoids the unwanted side‐effect(s) of DSF caused by other DSF's metabolites, such as the extreme sensitivity to any alcohol‐based drugs or disinfectants due to inhibition of aldehyde dehydrogenase [11], and it is patentable like any new compounds, thereby providing a greater incentive for further development by the pharmaceutical industry. In other words, we propose that effective, newly identified metabolites of approved drugs, such as CuET, combine the desirable features of drug repurposing and patentability without at least some drawbacks of the (pro)drug or the extreme costs and uncertainty involved in screening and developing entirely new compounds.

## 5 Conclusions

Our study provides a series of new preclinical models of common human cancers suitable for mechanistic elucidation of, and combating, chemoresistance. This work also provides experimental evidence supporting the notion of CuET as a candidate anticancer drug potentially useful for treatment of a wide range of tumours, including the advanced stages resistant to standard‐of‐care therapy. The future use of CuET will depend on results of clinical trials and the advice from regulatory authorities. Furthermore, if approval of CuET is obtained for clinical use in oncology, it is likely that initially only a combination with some standard‐of‐care can be considered.

## Conflicts of interest

The authors declare no conflicts of interest.

## Author contributions

SA, TB, RS and JB conceived the project. SA, TB, JIB, MLB, ZS, MM and JB designed the experiments and analysed the results. SA, TB, CLH, JIB, MLB, ZM and ED performed the experiments and acquired the data. JBL, BJM and MAS collected the tumour samples and performed the patient selection and logistics. MB performed the disease diagnostics, CH, EH and THS were involved in clinical logistics and data collection. TK supervised the studies involving patient‐derived tumour organoids. JB supervised the project. SA, TB and JB wrote the manuscript. JB, TK, AJ, SKK and RS provided the funding. All authors read and approved the manuscript and discussed and interpreted the data.

## Supporting information


**Fig. S1** Ratios of staining intensity for K48 ubiquitinated proteins in CuET/cisplatin‐treated/ and nontreated cells show unchanged responses to cisplatin and CuET of WT, compared with the more cisplatin resistance of cisR cells.
**Fig. S2.** K48 ubiquitinated proteins accumulates after CuET treatment in H1792 cells.
**Fig. S3.** K48 ubiquitinated proteins accumulate after CuET treatment in MDA‐MB‐231 cells.
**Fig. S4.** Ratio of staining intensity for γH2AX Ser139 in CuET/cisplatin‐treated/ and nontreated cells shows lower sensitivity to cisplatin response in four out of six resistant cell lines.
**Fig. S5.** γH2AX Ser139 levels increases after cisplatin in H1792 cells.
**Fig. S6.** γH2AX Ser139 levels increases after cisplatin in MDA‐MB‐231 cells.
**Fig. S7.** Ratio of staining intensity for DNA‐cisplatin adducts in CuET/cisplatin‐treated and nontreated cells shows the same or partially reduced adduct levels in cisR cells compared with. their WT counterparts.
**Fig. S8.** DNA‐cisplatin adducts are present after cisplatin treatment in H1792 cells.
**Fig. S9.** DNA‐cisplatin adducts are present after cisplatin treatment in MDA‐MB‐231 cells.
**Fig. S10.** CuET, but not cisplatin exposure evokes upregulation of *MT2A* and *MT1E* expression in WT and cisR LUAD cell lines.
**Fig. S11.** siRNA‐mediated depletion of MTF1 (siRNA #1) is followed by decreased metallothionein protein level and increased sensitivity of cells against CuET.
**Fig. S12.** Kaplan–Meier survival curves for cohorts of cancer patients in relation to metallothionein expression shows inverse correlation with the overall survival.

## Data Availability

Cell lines, organoids and data produced and used in this project are available upon request from the authors.

## References

[1] Romani AMP . Cisplatin in cancer treatment. Biochem Pharmacol. 2022;206:115323. 10.1016/j.bcp.2022.115323 36368406

[2] Manohar S , Leung N . Cisplatin nephrotoxicity: a review of the literature. J Nephrol. 2018;31(1):15–25. 10.1007/s40620-017-0392-z 28382507

[3] Bruno PM , Liu Y , Park GY , Murai J , Koch CE , Eisen TJ , et al. A subset of platinum‐containing chemotherapeutic agents kills cells by inducing ribosome biogenesis stress. Nat Med. 2017;23(4):461–471. 10.1038/nm.4291 PMC5520548 28263311

[4] Zisi A , Bartek J , Lindstrom MS . Targeting ribosome biogenesis in cancer: lessons learned and way forward. Cancers (Basel). 2022;14(9):2126. 10.3390/cancers14092126 PMC9100539 35565259

[5] Kaur R , Bhardwaj A , Gupta S . Cancer treatment therapies: traditional to modern approaches to combat cancers. Mol Biol Rep. 2023;50(11):9663–9676. 10.1007/s11033-023-08809-3 37828275

[6] Ramos A , Sadeghi S , Tabatabaeian H . Battling Chemoresistance in cancer: root causes and strategies to uproot them. Int J Mol Sci. 2021;22(17):9451. 10.3390/ijms22179451 PMC8430957 34502361

[7] Buchtova T , Beresova L , Chroma K , Pluhacek T , Beres T , Kaczorova D , et al. Cannabis‐derived products antagonize platinum drugs by altered cellular transport. Biomed Pharmacother. 2023;163:114801. 10.1016/j.biopha.2023.114801 37137184

[8] Lugones Y , Loren P , Salazar LA . Cisplatin resistance: genetic and epigenetic factors involved. Biomolecules. 2022;12(10):1365. 10.3390/biom12101365 PMC9599500 36291573

[9] Eslami M , Memarsadeghi O , Davarpanah A , Arti A , Nayernia K , Behnam B . Overcoming chemotherapy resistance in metastatic cancer: a comprehensive review. Biomedicine. 2024;12(1):183. 10.3390/biomedicines12010183 PMC10812960 38255288

[10] Kirtonia A , Gala K , Fernandes SG , Pandya G , Pandey AK , Sethi G , et al. Repurposing of drugs: an attractive pharmacological strategy for cancer therapeutics. Seminars in Cancer Biology. 2021;68:258–278. 10.1016/j.semcancer.2020.04.006 32380233

[11] Skrott Z , Majera D , Gursky J , Buchtova T , Hajduch M , Mistrik M , et al. Disulfiram's anti‐cancer activity reflects targeting NPL4, not inhibition of aldehyde dehydrogenase. Oncogene. 2019;38(40):6711–6722. 10.1038/s41388-019-0915-2 31391554

[12] Skrott Z , Mistrik M , Andersen KK , Friis S , Majera D , Gursky J , et al. Alcohol‐abuse drug disulfiram targets cancer via p97 segregase adaptor NPL4. Nature. 2017;552(7684):194–199. 10.1038/nature25016 PMC5730499 29211715

[13] Lanz J , Biniaz‐Harris N , Kuvaldina M , Jain S , Lewis K , Fallon BA . Disulfiram: mechanisms, applications, and challenges. Antibiotics. 2023;12(3):524. 10.3390/antibiotics12030524 PMC10044060 36978391

[14] Lu C , Li X , Ren Y , Zhang X . Disulfiram: a novel repurposed drug for cancer therapy. Cancer Chemother Pharmacol. 2021;87(2):159–172. 10.1007/s00280-020-04216-8 33426580

[15] Chroma K , Skrott Z , Gursky J , Bacovsky J , Moudry P , Buchtova T , et al. A drug repurposing strategy for overcoming human multiple myeloma resistance to standard‐of‐care treatment. Cell Death Dis. 2022;13(3):203. 10.1038/s41419-022-04651-w PMC8897388 35246527

[16] Kanellis DCDC , Kanellis DC , Zisi A , Skrott Z , Lemmens B , Espinoza JA , et al. Actionable cancer vulnerability due to translational arrest, p53 aggregation and ribosome biogenesis stress evoked by the disulfiram metabolite CuET. Cell Death Differ. 2023;30:1666–1678. 10.1038/s41418-023-01167-4 PMC10307793 37142656

[17] Majera D , Skrott Z , Chroma K , Merchut‐Maya JM , Mistrik M , Bartek J . Targeting the NPL4 adaptor of p97/VCP Segregase by disulfiram as an emerging cancer vulnerability evokes replication stress and DNA damage while silencing the ATR pathway. Cells. 2020;9(2):469. 10.3390/cells9020469 PMC7072750 32085572

[18] Feoktistova M , Geserick P , Leverkus M . Crystal violet assay for determining viability of cultured cells. Cold Spring Harb Protoc. 2016;2016(4):pdbprot087379. 10.1101/pdb.prot087379 27037069

[19] Buchtova T , Skrott Z , Chroma K , Rehulka J , Dzubak P , Hajduch M , et al. Cannabidiol‐induced activation of the metallothionein pathway impedes anticancer effects of disulfiram and its metabolite CuET. Mol Oncol. 2022;16(7):1541–1554. 10.1002/1878-0261.13114 PMC8978514 34632694

[20] Kosar M , Giannattasio M , Piccini D , Maya‐Mendoza A , García‐Benítez F , Bartkova J , et al. The human nucleoporin Tpr protects cells from RNA‐mediated replication stress. Nat Commun. 2021;12(1):3937. 10.1038/s41467-021-24224-3 PMC8225803 34168151

[21] Gogola E , Duarte AA , Ruiter JR , Wiegant WW , Schmid JA , Bruijn R , et al. Selective loss of PARG restores PARylation and counteracts PARP inhibitor‐mediated synthetic lethality. Cancer Cell. 2018;33(6):1078–1093.e12. 10.1016/j.ccell.2018.05.008 29894693

[22] Bouwman P , Aly A , Escandell JM , Pieterse M , Bartkova J , van der Gulden H , et al. 53BP1 loss rescues BRCA1 deficiency and is associated with triple‐negative and BRCA‐mutated breast cancers. Nat Struct Mol Biol. 2010;17(6):688–695. 10.1038/nsmb.1831 PMC2912507 20453858

[23] Paes Dias M , Tripathi V , van der Heijden I , Cong K , Manolika EM , Bhin J , et al. Loss of nuclear DNA ligase III reverts PARP inhibitor resistance in BRCA1/53BP1 double‐deficient cells by exposing ssDNA gaps. Mol Cell. 2021;81(22):4692–4708.e9. 10.1016/j.molcel.2021.09.005 PMC9098260 34555355

[24] Liu J , Zheng Y , Xiong Y , Ma R , Lin Z , Lin H , et al. Nucleoporin TPR integrates MAPK signaling with mitogen‐induced transcriptional programs. Cell Death Dis. 2026;17(1):400. 10.1038/s41419-026-08760-8 PMC13109368 42031697

[25] Bartkova J , Hořejší Z , Koed K , Krämer A , Tort F , Zieger K , et al. DNA damage response as a candidate anti‐cancer barrier in early human tumorigenesis. Nature. 2005;434(7035):864–870. 10.1038/nature03482 15829956

[26] Goulding H , Jasani B , Pereira H , Reid A , Galea M , Bell JA , et al. Metallothionein expression in human breast cancer. Br J Cancer. 1995;72(4):968–972. 10.1038/bjc.1995.443 PMC2034037 7547250

[27] Skorda A , Lauridsen AR , Huhtinen K , Lahtinen A , Senkowski W , Oikkonen J , et al. Quantification of cell death and proliferation of patient‐derived ovarian cancer organoids through 3D imaging and image analysis. STAR Protoc. 2023;4(4):102683. 10.1016/j.xpro.2023.102683 PMC10692951 37976153

[28] Bañuelos CA , Banáth JP , Kim JY , Aquino‐Parsons C . γH2AX expression in tumors exposed to cisplatin and fractionated irradiation. Clin Cancer Res. 2009;15(10):3344–3353. 10.1158/1078-0432.CCR-08-3114 19401347

[29] Liedert B , Pluim D , Schellens J , Thomale J . Adduct‐specific monoclonal antibodies for the measurement of cisplatin‐induced DNA lesions in individual cell nuclei. Nucleic Acids Res. 2006;34(6):e47. 10.1093/nar/gkl051 PMC1420801 16571898

[30] Strauss R , Li ZY , Liu Y , Beyer I , Persson J , Sova P , et al. Analysis of epithelial and mesenchymal markers in ovarian cancer reveals phenotypic heterogeneity and plasticity. PLoS One. 2011;6(1):e16186. 10.1371/journal.pone.0016186 PMC3021543 21264259

[31] Strauss R , Sova P , Liu Y , Li ZY , Tuve S , Pritchard D , et al. Epithelial phenotype confers resistance of ovarian cancer cells to oncolytic adenoviruses. Cancer Res. 2009;69(12):5115–5125. 10.1158/0008-5472.CAN-09-0645 PMC2738419 19491256

[32] Grosse‐Wilde A , Fouquier d'Hérouël A , McIntosh E , Ertaylan G , Skupin A , Kuestner RE , et al. Stemness of the hybrid epithelial/mesenchymal state in breast cancer and its association with poor survival. PLoS One. 2015;10(5):e0126522. 10.1371/journal.pone.0126522 PMC4447403 26020648

[33] Perez‐Gonzalez A , Bevant K , Blanpain C . Cancer cell plasticity during tumor progression, metastasis and response to therapy. Nat Can. 2023;4(8):1063–1082. 10.1038/s43018-023-00595-y PMC7615147 37537300

[34] Corsello SM , Nagari RT , Spangler RD , Rossen J , Kocak M , Bryan JG , et al. Discovering the anticancer potential of non‐oncology drugs by systematic viability profiling. Nat Can. 2020;1(2):235–248. 10.1038/s43018-019-0018-6 PMC7328899 32613204

[35] Merlos Rodrigo MA , Jimenez Jimemez AM , Haddad Y , Bodoor K , Adam P , Krizkova S , et al. Metallothionein isoforms as double agents ‐ their roles in carcinogenesis, cancer progression and chemoresistance. Drug Resist Updat. 2020;52:100691. 10.1016/j.drup.2020.100691 32615524

[36] Alonso‐Gonzalez C , Mediavilla D , Martinez‐Campa C , Gonzalez A , Cos S , Sanchez‐Barcelo EJ . Melatonin modulates the cadmium‐induced expression of MT‐2 and MT‐1 metallothioneins in three lines of human tumor cells (MCF‐7, MDA‐MB‐231 and HeLa). Toxicol Lett. 2008;181(3):190–195. 10.1016/j.toxlet.2008.07.021 18755257

[37] Borchert S , Suckrau PM , Walter RFH , Wessolly M , Mairinger E , Steinborn J , et al. Impact of metallothionein‐knockdown on cisplatin resistance in malignant pleural mesothelioma. Sci Rep. 2020;10(1):18677. 10.1038/s41598-020-75807-x PMC7596082 33122816

[38] Hou XF , Fan QX , Wang LX , Lu SX . Role of metallothionein1h in cisplatin resistance of non‐small cell lung cancer cells. Chin J Cancer Res. 2009;21(4):247–254.

[39] Si M , Lang J . The roles of metallothioneins in carcinogenesis. J Hematol Oncol. 2018;11(1):107. 10.1186/s13045-018-0645-x PMC6108115 30139373

[40] Takahashi S . Positive and negative regulators of the metallothionein gene (review). Mol Med Rep. 2015;12(1):795–799. 10.3892/mmr.2015.3459 25760317

[41] Győrffy B . Integrated analysis of public datasets for the discovery and validation of survival‐associated genes in solid tumors. Innovation (N Y). 2024;5(3):100625. 10.1016/j.xinn.2024.100625 PMC11066458 38706955

[42] Lánczky A , Győrffy B . Web‐based survival analysis tool tailored for medical research (KMplot): development and implementation. J Med Internet Res. 2021;23(7):e27633. 10.2196/27633 PMC8367126 34309564

[43] Zhang C , Xu C , Gao X , Yao Q . Platinum‐based drugs for cancer therapy and anti‐tumor strategies. Theranostics. 2022;12(5):2115–2132. 10.7150/thno.69424 PMC8899578 35265202

[44] Nechushtan H , Hamamreh Y , Nidal S , Gotfried M , Baron A , Shalev YI , et al. A phase IIb trial assessing the addition of disulfiram to chemotherapy for the treatment of metastatic non‐small cell lung cancer. Oncologist. 2015;20(4):366–367. 10.1634/theoncologist.2014-0424 PMC4391770 25777347

