# Supplementary material for: Overcoming platinum resistance by the disulfiram metabolite CuET in novel preclinical models of human carcinomas
Source: Mol Oncol. 2026 Nov 4:10.1002/1878-0261.70356. Online ahead of print. doi: 10.1002/1878-0261.70356 (PMC13732508; doi:10.1002/1878-0261.70356)
Supplement: Supplementary file 1 [file MOL2-9999-0-s001.pdf]

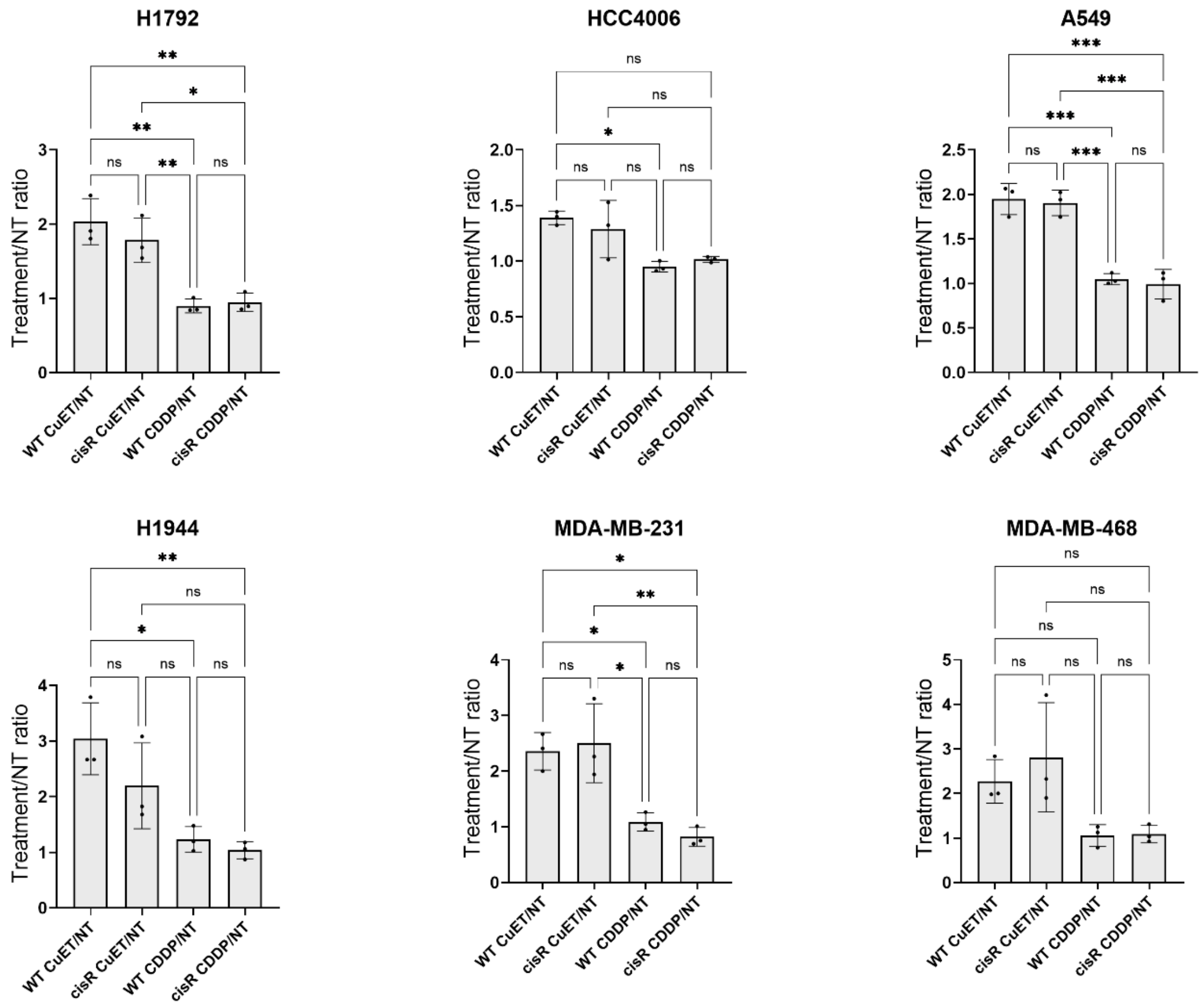

Supplementary Figure 1: **Ratios of staining intensity for K48 ubiquitinated proteins in CuET/cisplatin -treated/ and non-treated cells shows unchanged responses to cisplatin and CuET of WT, compared to the more cisplatin resistance of ciscisR cells.** Cells were treated with 1μM CuET and 20 μM cisplatin (CDDP) for 4 h. Cisplatin internalization was evaluated via CDDP-adducts staining. Samples were analyzed in a scanR High-Content Screening Station. The ratio of the average of the mean intensity K48 ubiquitinated proteins antibody in CuET/CDDP-treated cells/control cells for each WT and cisR cell line is presented. Statistical significance was assessed with Ordinary one-way ANOVA. $P<0.0001$****, $P<0.001$***, $P<0.01$**, $P<0.05$*, ns = non-significant, NT = non-treated. Data shown as mean with SD, n=3.

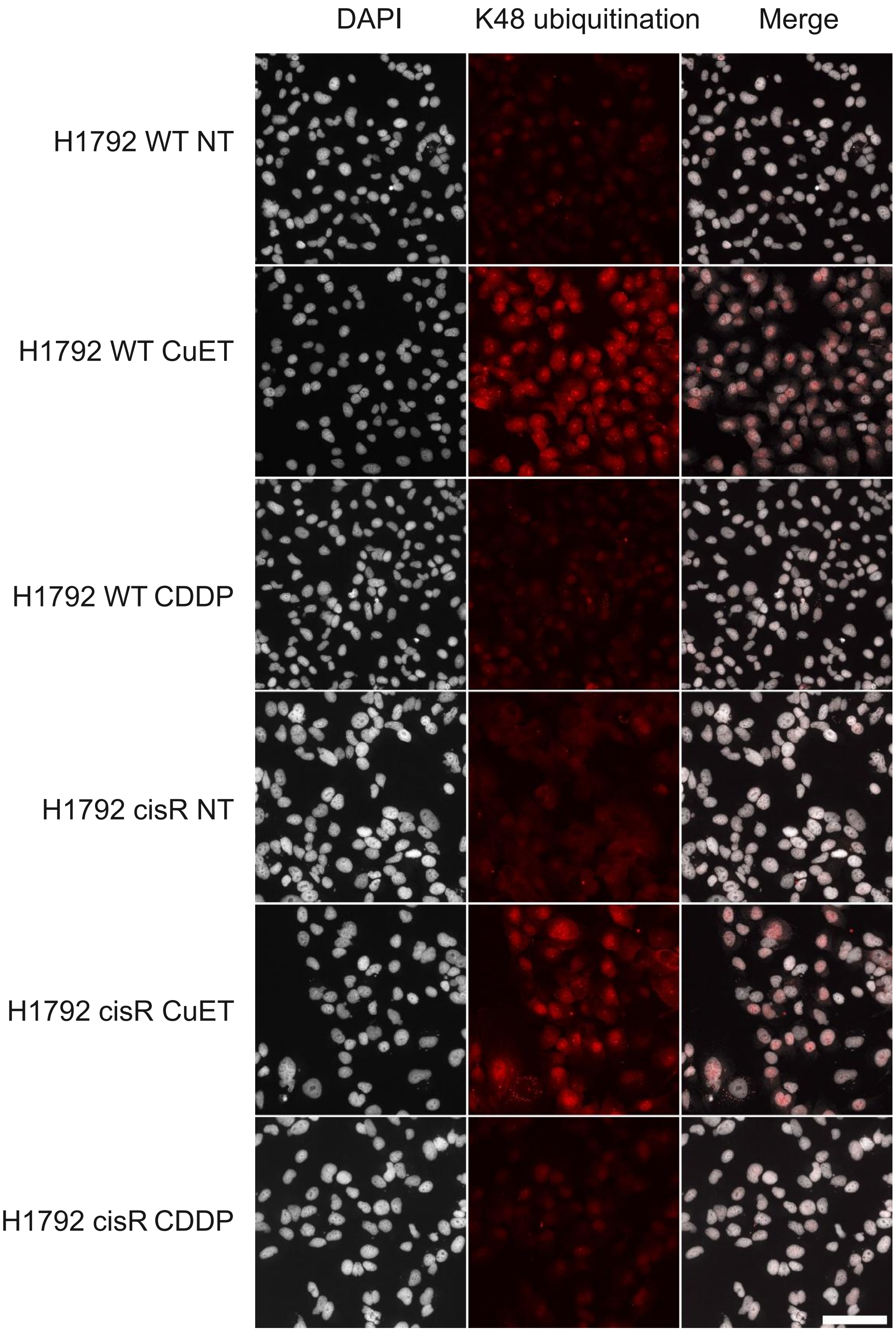

Supplementary Figure 2: **K48 ubiquitinated proteins accumulatesion after CuET treatment in H1792 cells.** Representative immunofluorescence (IF) microscopy images of H1792 WT and H1792 cisR cells. Cells were treated with 1μM CuET and 20 μM cisplatin (CDDP) for 4 h. IF staining was performed with a K48 ubiquitinated proteins (red) antibody. Nuclei were counterstained with DAPI (white). Samples were analyzed in a scanR High-Content Screening Station. Bar=100 μm, NT = non-treated.

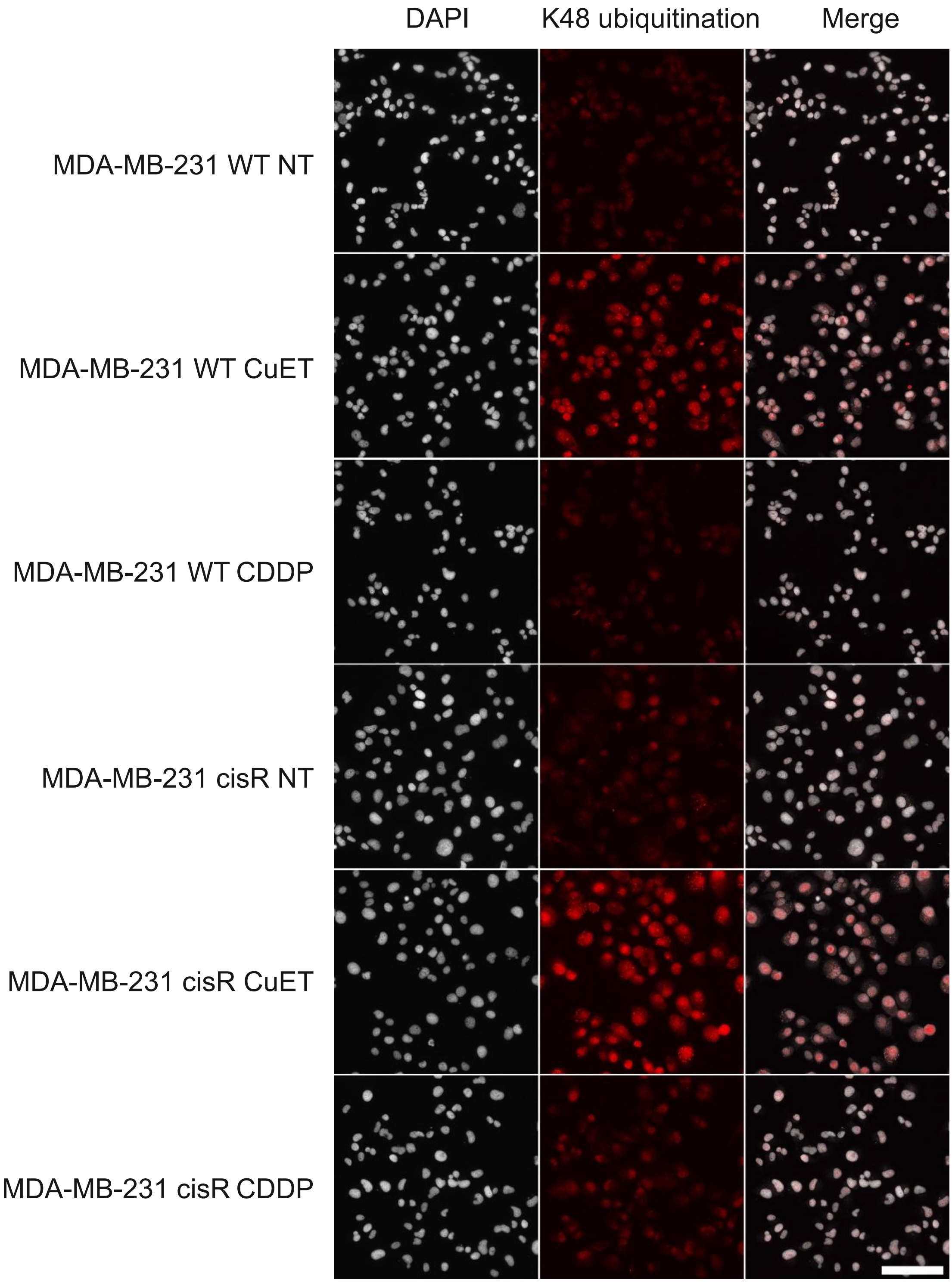

Supplementary Figure 3: **K48 ubiquitinated proteins accumulates after CuET treatmention in MDA-MB-231 cells.** Representative microscopy images of MDA-MB-231 WT and MDA-MB-231 cisR cells. Cells were treated with 1µM CuET and 20 µM cisplatin (CDDP), respectively, for 4 h. IF analysis was performed with an antibody against K48 ubiquitinated proteins (red) antibody. Nuclei were counterstained with DAPI (white). Samples were analyzed in a scanR High-Content Screening Station. Bar=100 µm, NT = non-treated.

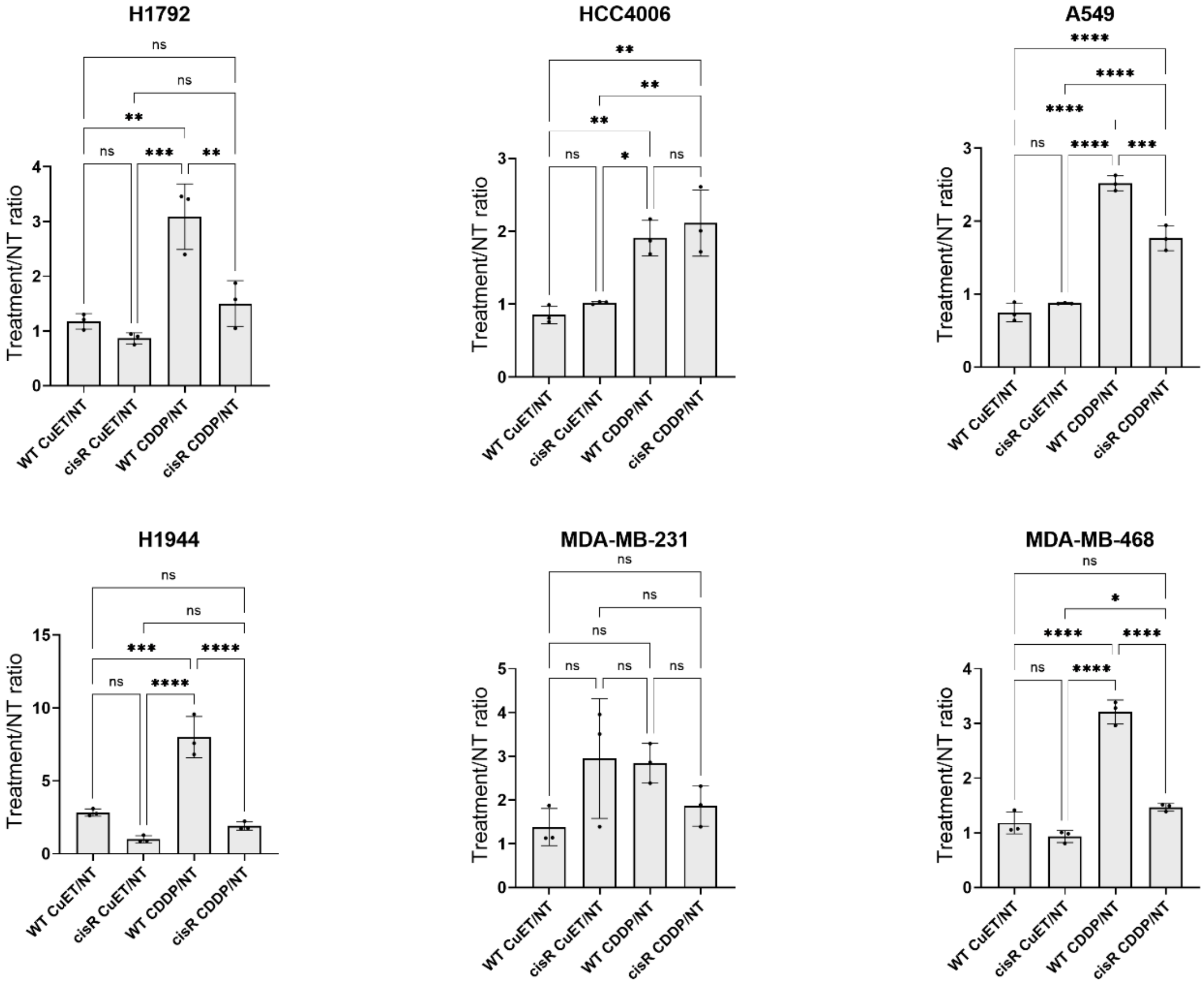

Supplementary Figure 4: **Ratio of staining intensity for γH2AX Ser139 in CuET/cisplatin -treated/ and non-treated cells shows lower sensitivity to cisplatin response in 4 out of 6 resistant cell lines.** Cells were treated with 1μM CuET and 20 μM cisplatin (CDDP), respectively, for 4 h. Cisplatin internalization was evaluated via CDDP-adduct staining. Samples were analyzed in a scanR High-Content Screening Station. The ratio of the average of the mean intensity γH2AX Ser139 antibody in CuET/CDDP-treated cells/control cells for each WT and cisR cell line is presented. Statistical significance was assessed with Ordinary one-way ANOVA. P<0.0001****, P<0.001***, P<0.01**, P<0.05*, ns = non-significant, NT = non-treated. Data shown as mean with SD, n=3.

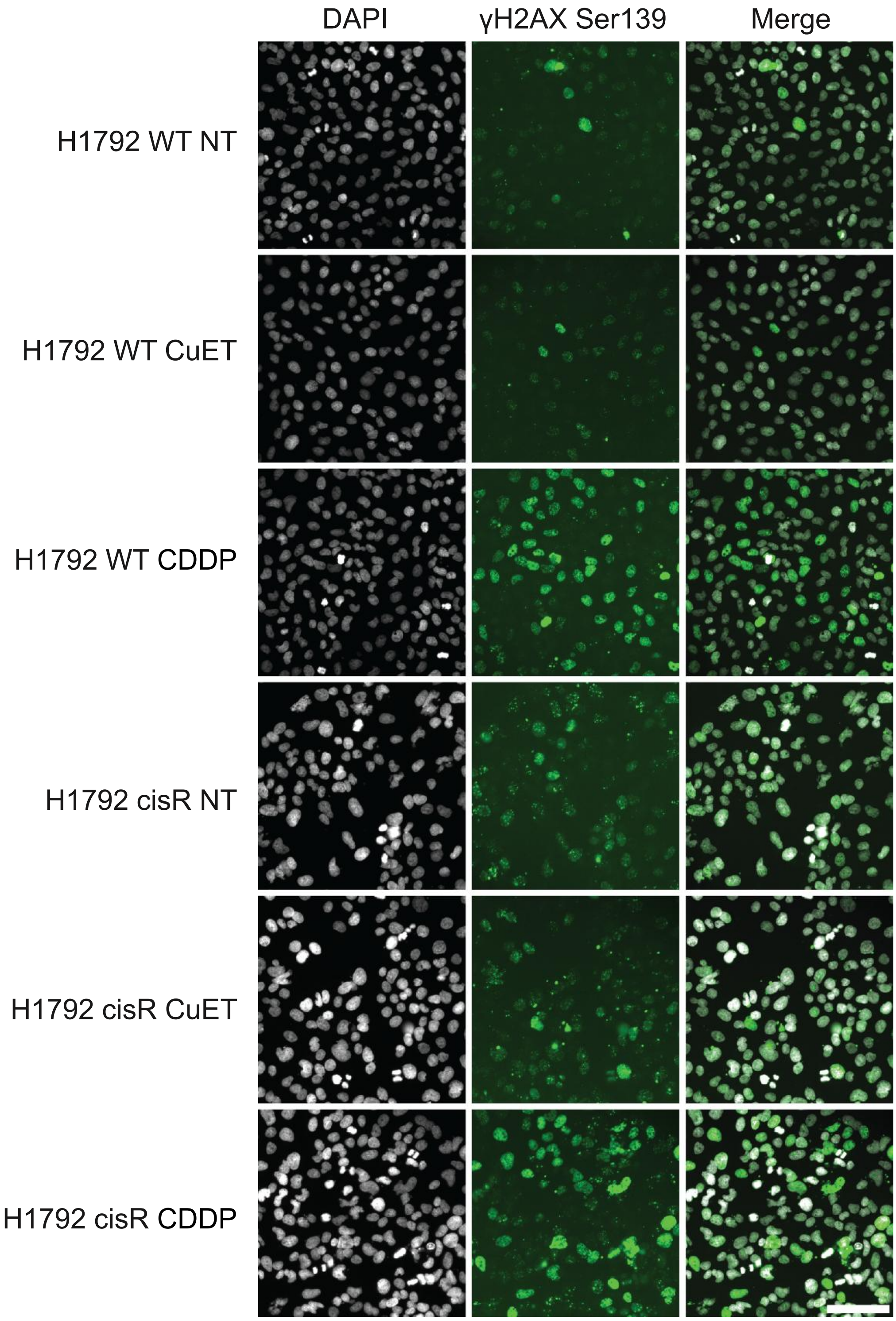

Supplementary Figure 5: **γH2AX Ser139 levels increases after cisplatin in H1792 cells.** Representative immunofluorescence microscopy images of H1792 WT and H1792 cisR cells. Cells were treated with 1µM CuET and 20 µM cisplatin (CDDP) for 4 h. IF analysis was performed with a γH2AX Ser139 (green) antibody. Nuclei were counterstained with DAPI (white). Samples were analyzed in a scanR High-Content Screening Station. Bar=100 µm, NT = non-treated.

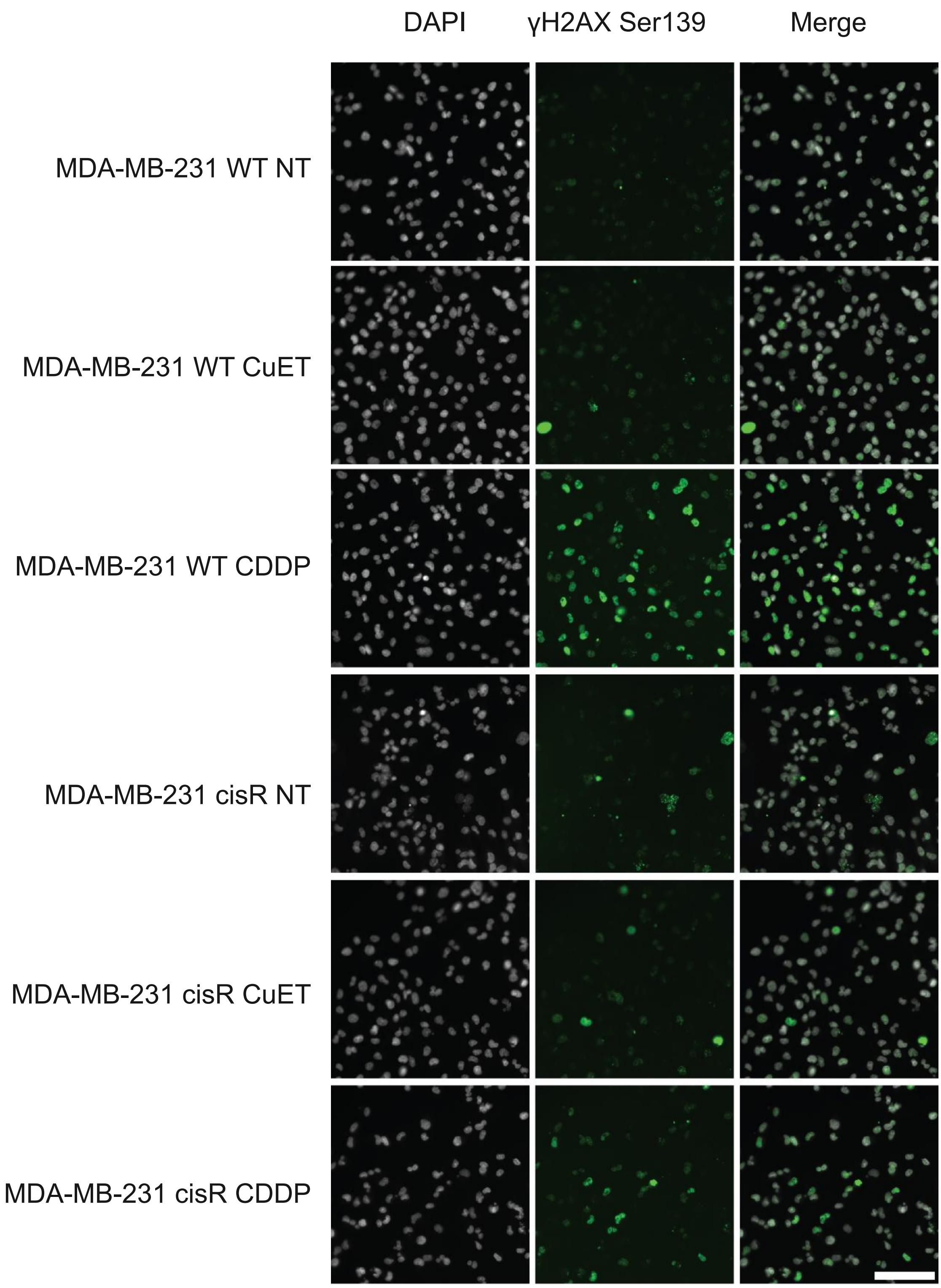

Supplementary Figure 6: **γH2AX Ser139 levels increases after cisplatin in MDA-MB-231 cells.** Representative microscopy images of MDA-MB-231 WT and MDA-MB-231 cisR cells. Cells were treated with 1μM CuET and 20 μM cisplatin (CDDP), respectively, for 4 h. IF analysis was performed with a γH2AX Ser139 (green) antibody. Nuclei were counterstained with DAPI (white). Samples were analyzed in a scanR High-Content Screening Station. Bar=100 μm, NT = non-treated.

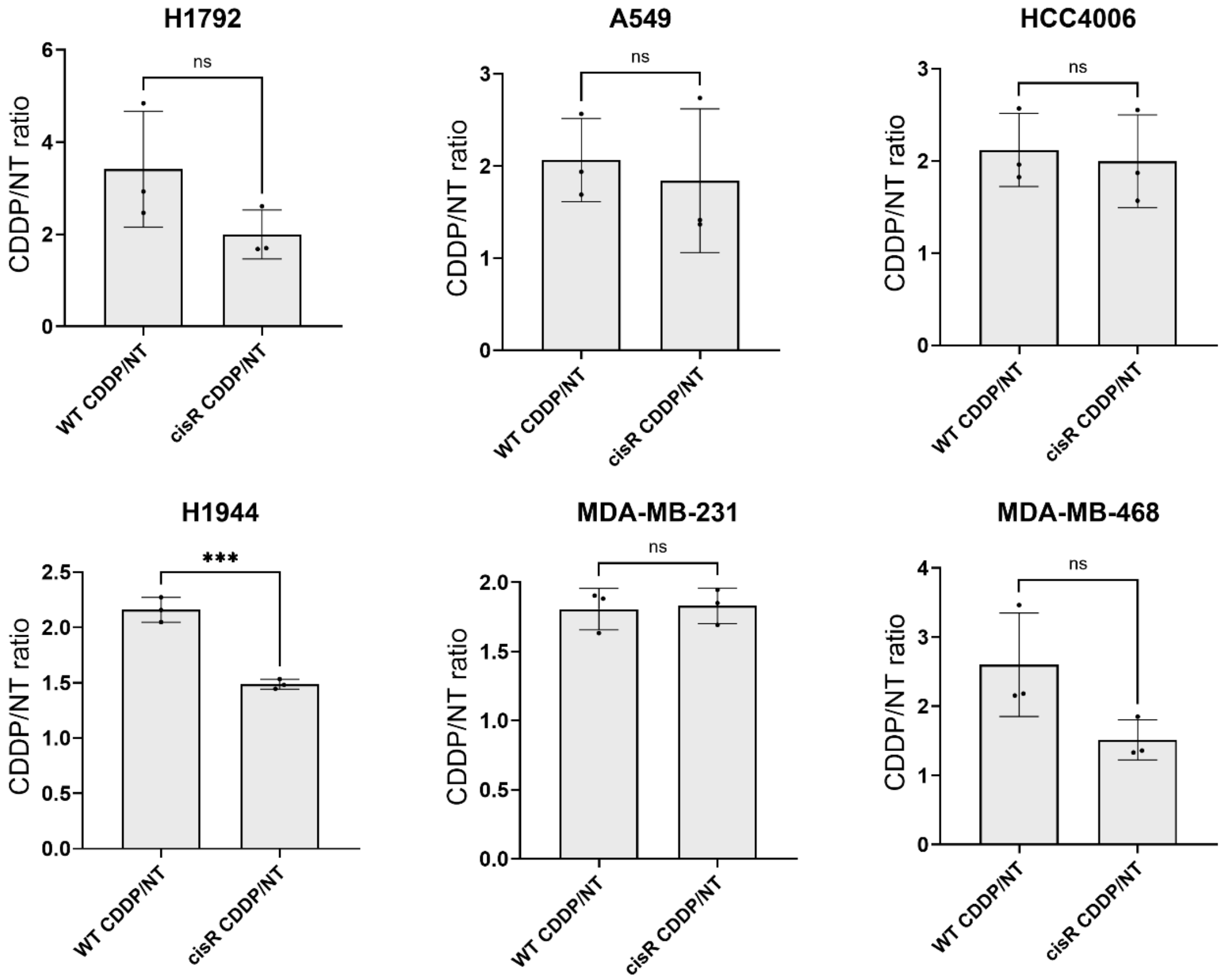

Supplementary Figure 7: **Ratio of staining intensity for DNA-cisplatin adducts in CuET/cisplatin -treated and non-treated cells shows the same or partially reduced adduct levels in cisR cells compared to their WT counterparts.** Cells were treated with 1µM CuET and 20 µM cisplatin (CDDP), respectively, for 4 h. Cisplatin internalization was evaluated indirectly via CDDP-adducts staining. Samples were analyzed in a scanR High-Content Screening Station. The ratio of the average of the mean intensity K48 ubiquitinated proteins antibody in CuET/CDDP-treated cells/control cells for each WT and cisR cell line is presented. Statistical significance was assessed with Ordinary one-way ANOVA. $P<0.0001$****, $P<0.001$***, $P<0.01$**, $P<0.05$*, ns = non-significant, NT = non-treated. Data shown as mean with SD, n=3.

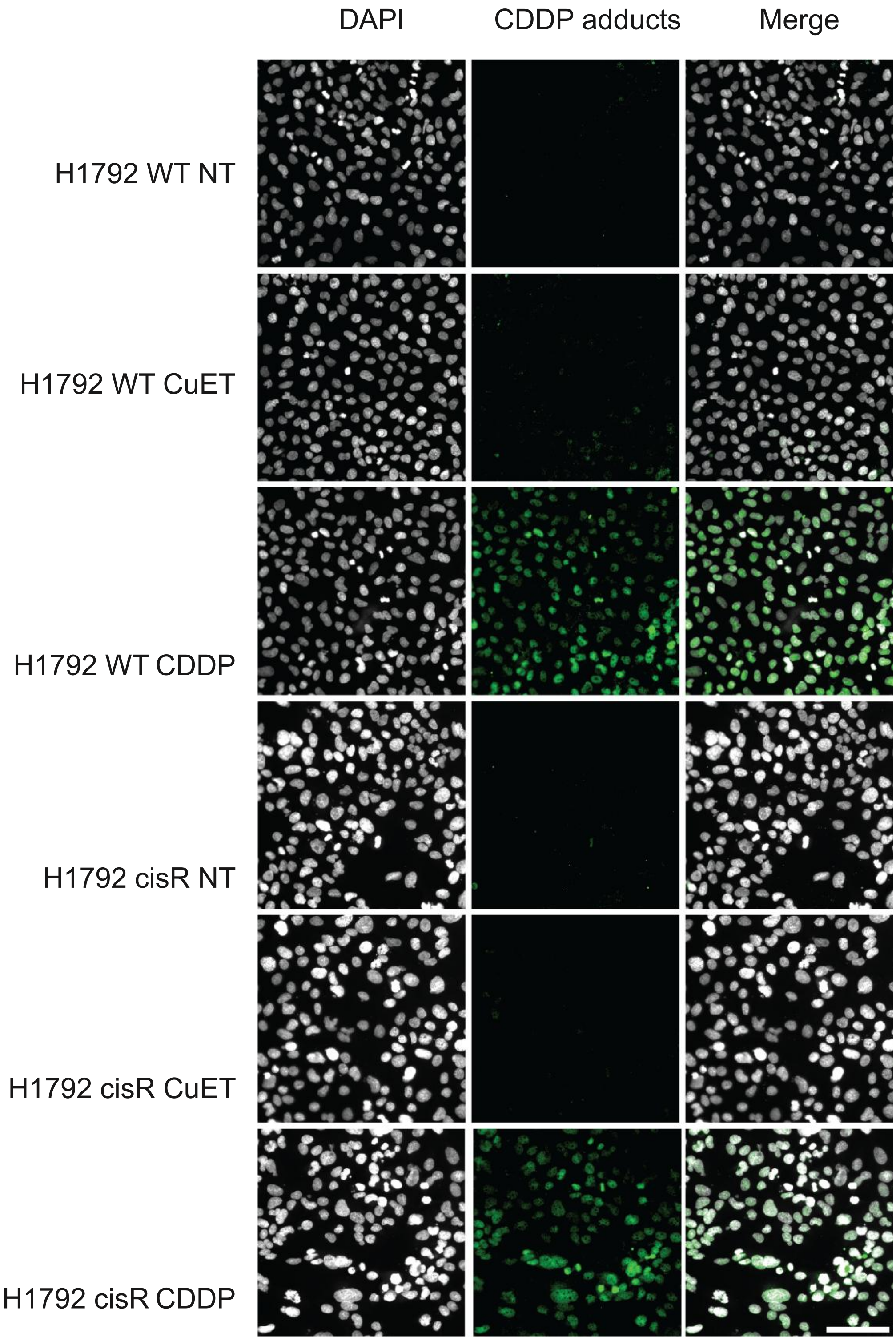

Supplementary Figure 8: **DNA-cisplatin adducts are present after cisplatin treatment in H1792 cells.**Representative immunofluorescence microscopy images of H1792 WT and H1792 cisR cells. Cells were treated with 1μM CuET and 20 μM cisplatin (CDDP), respectively, for 4 h. IF analysis was performed with a DNA-cisplatin adducts (red) antibody. Nuclei were counterstained with DAPI (white). Samples were analyzed in a scanR High-Content Screening Station. Bar=100 μm, NT = non-treated.

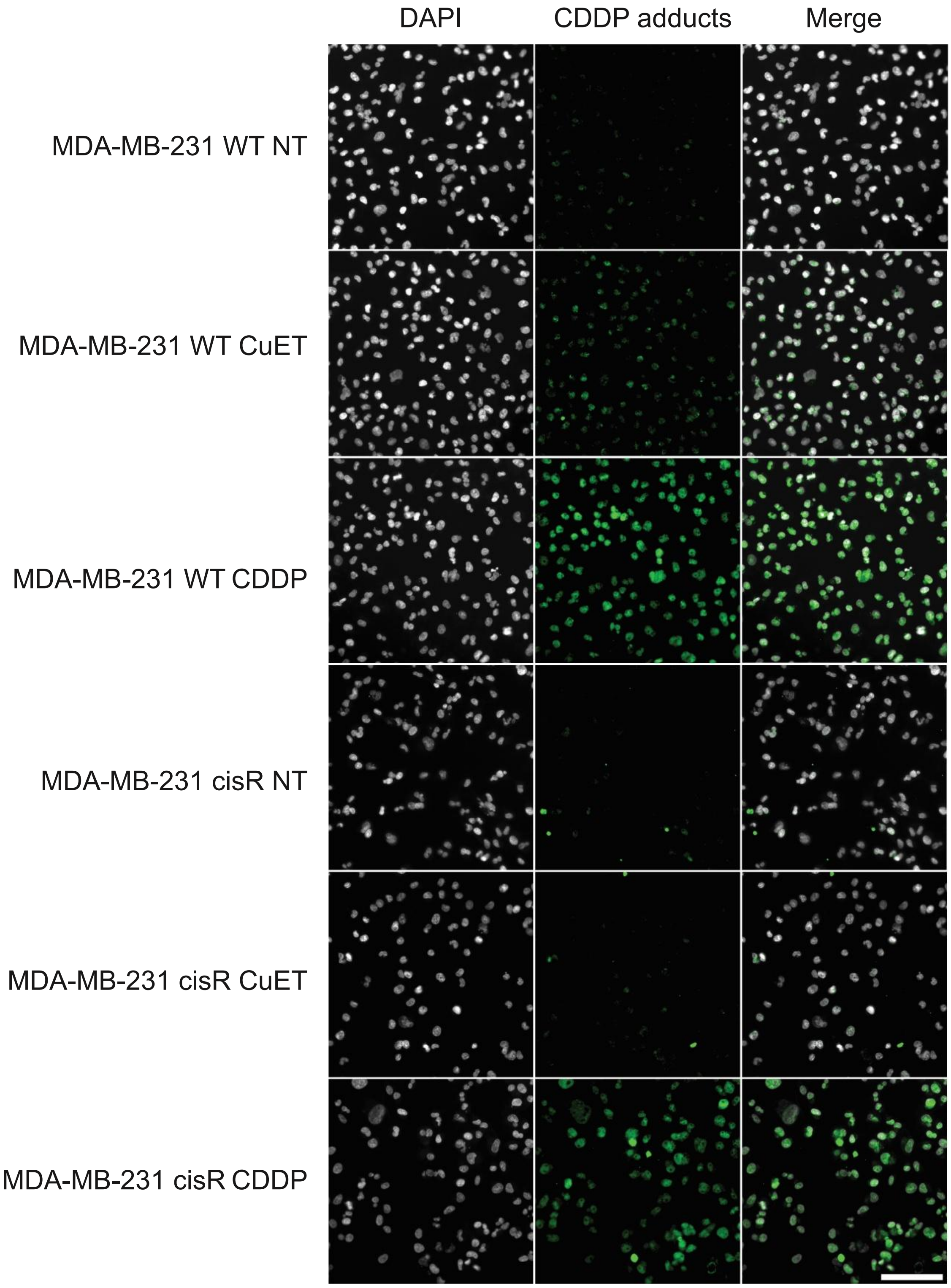

Supplementary Figure 9: **DNA-cisplatin adducts are present after cisplatin treatment in MDA-MB-231 cells.** Representative immunofluorescence microscopy images of MDA-MB-231 WT and MDA-MB-231 cisR cells. Cells were treated with 1µM CuET and 20 µM cisplatin (CDDP), respectively, for 4 h. IF analysis was performed with a DNA-cisplatin adducts (red) antibody. Nuclei were counterstained with DAPI (white). Samples were analyzed in a scanR High-Content Screening Station. Bar=100 µm, NT = non-treated.

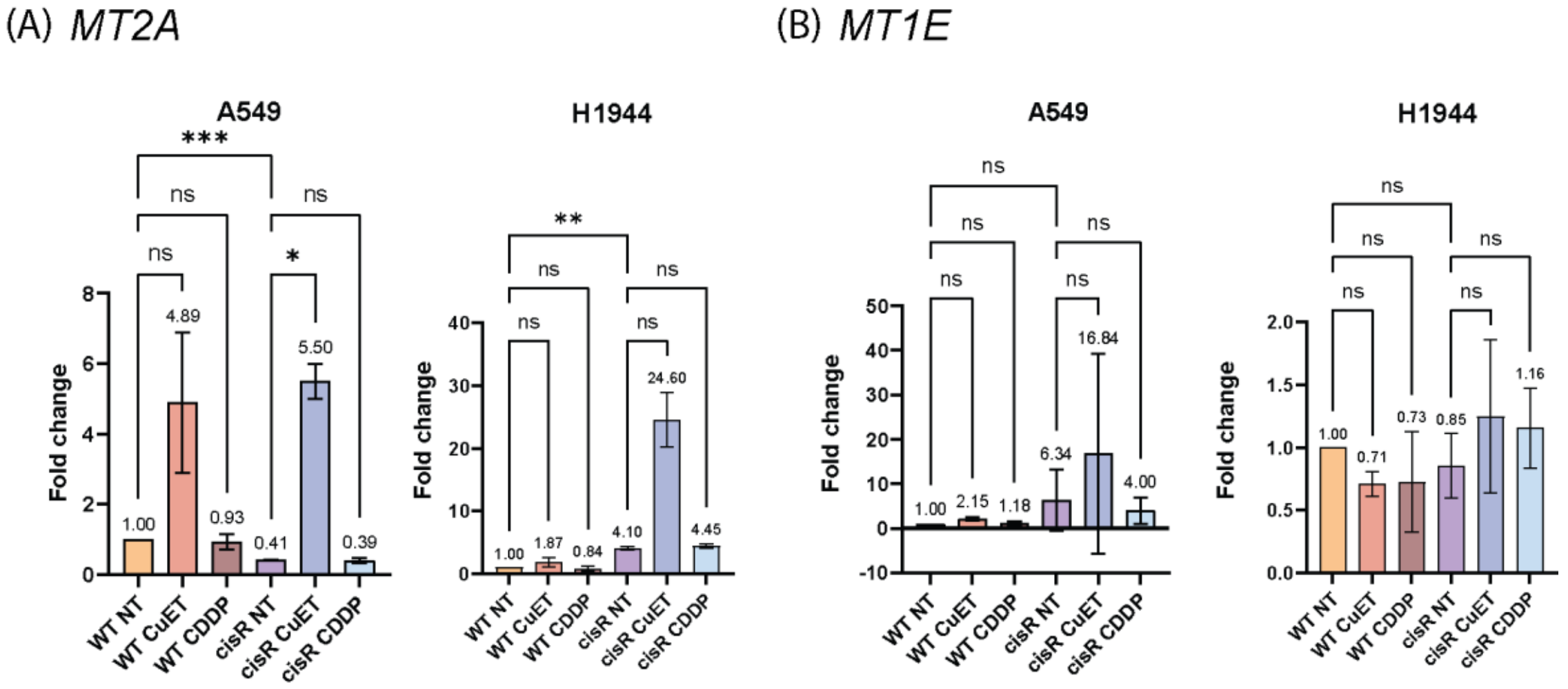

Supplementary Figure 10: **CuET, but not cisplatin exposure evokes upregulation of MT2A and MT1E expression in WT and cisR LUAD cell lines.** The expression levels of A) *MT2A*, and B) *MT1E* were evaluated via qPCR. Cells were treated with 1 µM CuET and 20 µM cisplatin (CDDP) for 4 h. Statistical significance was assessed with the one-way ANOVA test. P<0.0001****, P<0.001***, P<0.01**, P<0.05*, ns = non-significant, NT = non-treated. Data shown as mean with SD, n=3.

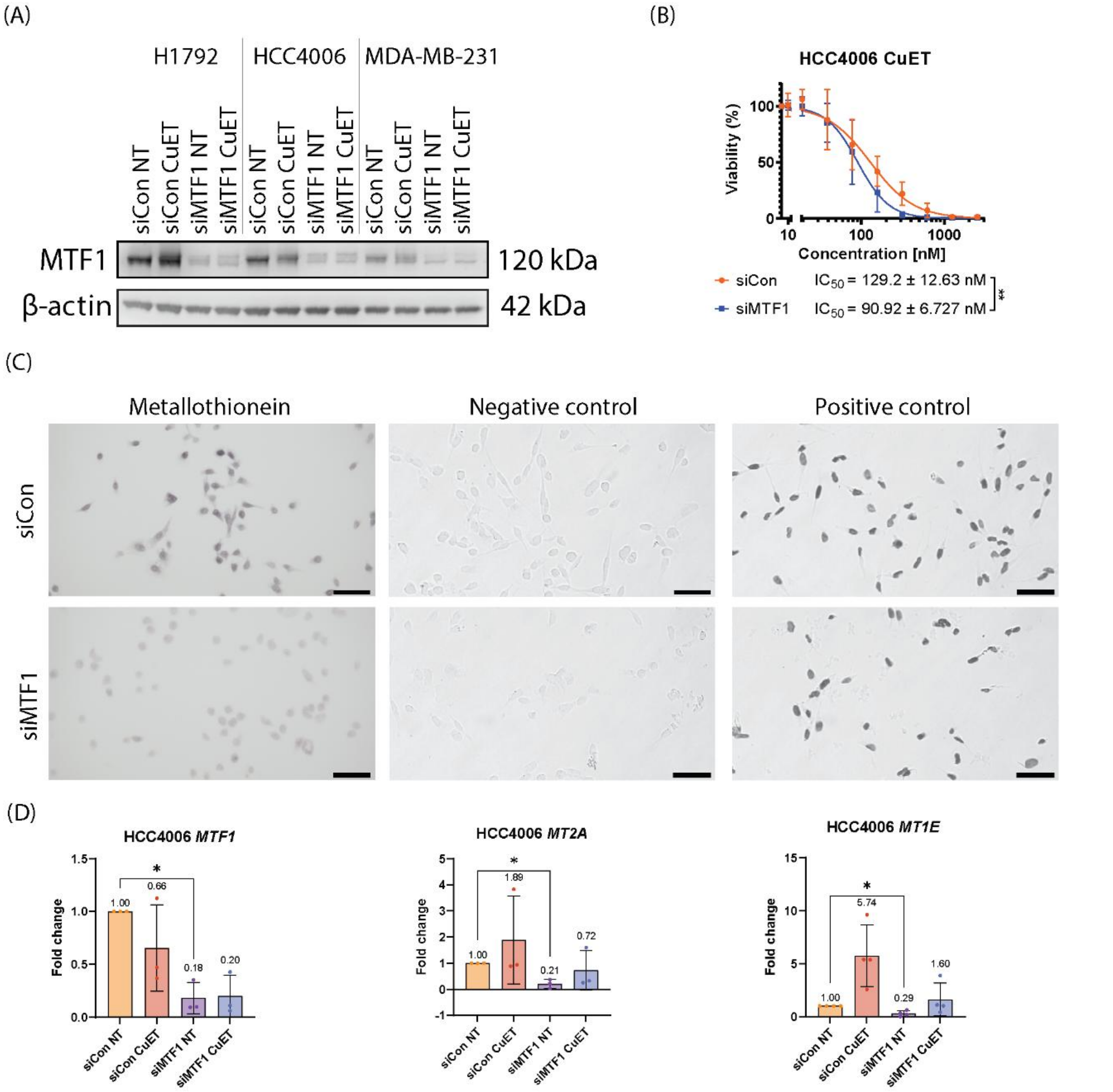

Supplementary Figure 11: **siRNA-mediated depletion of MTF1 (siRNA #1) is followed by decreased metallothionein protein level and increased sensitivity of cells against CuET.** A) Western blotting verification of MTF1 knockdown. 3 days after the knockdown, cells were treated with 1µM CuET for 4 hours. n=3. B) 24 h after knockdown, HCC4006 WT cells were plated in 96-well plates. 48 h later they were treated with a range of CuET (0-2,500 nM) concentrations. Following 96 h of incubation, the plates were stained with Hoechst and SYTOX™ Deep Red analyzed in a scanR High-Content Screening Station. n=4. Statistically significant differences between curve fits were evaluated by sum-of-squares F-test: P<0.0001****, P<0.001***, P<0.01**, P<0.05*, ns = non-significant, IC50±SE. C) 24 h after knockdown, MDA-MB-231 WT cells were plated in 6-well plates. 48 h later the metallothionein expression was evaluated via immunocytochemistry. Negative and positive controls (Nucleoporin TPR) are described in Methods (section 2.6). Scale bar = 50 µm. D) 24 h after knockdown, HCC4006 WT cells were plated in 6-well plates. 48 h later they were treated with 1µM CuET. Following 4 h of incubation the expression levels of *MTF1, MT2A* and *MT1E* were evaluated *via* qPCR. Statistical significance was assessed with the one-way ANOVA test. P<0.0001****, P<0.001***, P<0.01**, P<0.05*, ns = non-significant, NT = non-treated. Data shown as mean with SD, n=3.

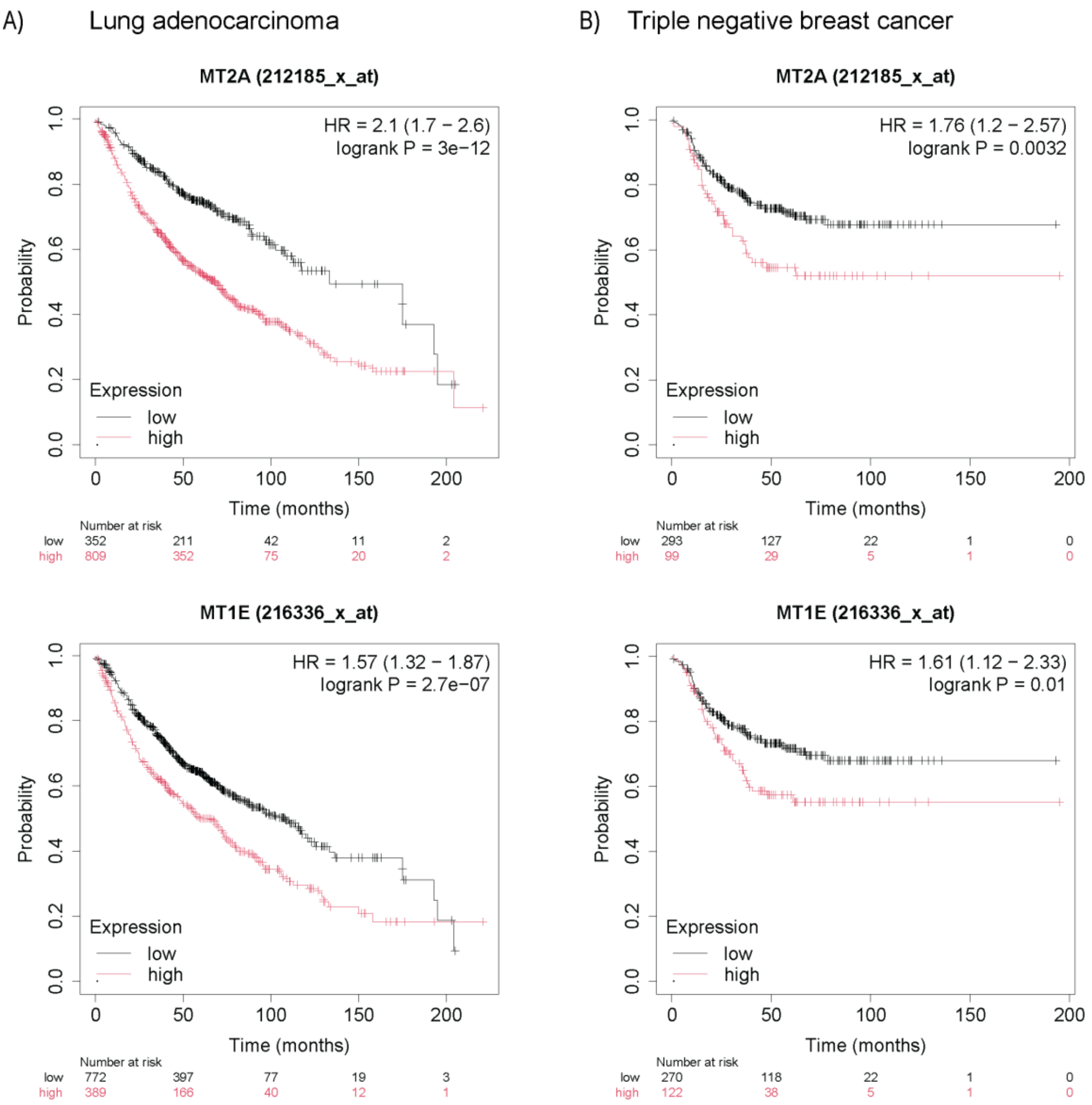

Supplementary Figure 12: **Kaplan-Meier survival curves for cohorts of cancer patients in relation to metallothionein expression shows inverse corelation with the overall survival.** The Kaplan-Meier survival curves were generated using available repository data for survival times of patients with high (red line) and low (black line) expression groups. Differences between the survival curves were assessed using the log-rank test. The analysis was performed using the kmplot website, and the tumor types are indicated in the individual panels. See Methods for more details.
